# An efficient CRISPR/Cas9 genome editing system based on a multiple sgRNA processing platform in *Trichoderma reesei* for strain improvement and enzyme production

**DOI:** 10.1186/s13068-024-02468-7

**Published:** 2024-02-11

**Authors:** Jiaxin Zhang, Kehang Li, Yu Sun, Cheng Yao, Weifeng Liu, Hong Liu, Yaohua Zhong

**Affiliations:** State Key Laboratory of Microbial Technology, Institute of Microbial Technology, Shandong University, Qingdao, 266237 People’s Republic of China

**Keywords:** CRISPR/Cas9, *Trichoderma reesei*, Multigene editing, Glucose oxidase, Lignocellulose-degrading enzymes, Multiple sgRNA processing

## Abstract

**Background:**

The CRISPR/Cas9 technology is being employed as a convenient tool for genetic engineering of the industrially important filamentous fungus *Trichoderma reesei*. However, multiplex gene editing is still constrained by the sgRNA processing capability, hindering strain improvement of *T. reesei* for the production of lignocellulose-degrading enzymes and recombinant proteins.

**Results:**

Here, a CRISPR/Cas9 system based on a multiple sgRNA processing platform was established for genome editing in *T. reesei*. The platform contains the arrayed tRNA−sgRNA architecture directed by a *5S rRNA* promoter to generate multiple sgRNAs from a single transcript by the endogenous tRNA processing system. With this system, two sgRNAs targeting *cre1* (encoding the carbon catabolite repressor 1) were designed and the precise deletion of *cre1* was obtained, demonstrating the efficiency of sgRNAs processing in the tRNA−sgRNA architecture. Moreover, overexpression of *xyr1-A824V* (encoding a key activator for cellulase/xylanase expression) at the *ace1* (encoding a repressor for cellulase/xylanase expression) locus was achieved by designing two sgRNAs targeting *ace1* in the system, resulting in the significantly enhanced production of cellulase (up to 1- and 18-fold on the Avicel and glucose, respectively) and xylanase (up to 11- and 41-fold on the Avicel and glucose, respectively). Furthermore, heterologous expression of the glucose oxidase gene from *Aspergillus niger* ATCC 9029 at the *cbh1* locus with the simultaneous deletion of *cbh1* and *cbh2* (two cellobiohydrolase coding genes) by designing four sgRNAs targeting *cbh1* and *cbh2* in the system was acquired, and the glucose oxidase produced by *T. reesei* reached 43.77 U/mL. Besides, it was found the ER-associated protein degradation (ERAD) level was decreased in the glucose oxidase-producing strain, which was likely due to the reduction of secretion pressure by deletion of the major endogenous cellulase-encoding genes.

**Conclusions:**

The tRNA−gRNA array-based CRISPR-Cas9 editing system was successfully developed in *T. reesei.* This system would accelerate engineering of *T. reesei* for high-level production of enzymes including lignocellulose-degrading enzymes and other recombinant enzymes. Furthermore, it would expand the CRISPR toolbox for fungal genome editing and synthetic biology.

**Supplementary Information:**

The online version contains supplementary material available at 10.1186/s13068-024-02468-7.

## Background

The filamentous fungus *Trichoderma reesei* is an efficient producer for industrial production of lignocellulose-degrading enzymes, because of its brilliant protein secretion ability [1]. A full range of the secreted lignocellulolytic enzymes include cellobiohydrolase (CBH), endoglucanase (EG), β-glucosidase (BGL), xylanase (XYN), and lytic polysaccharide monooxygenase (LPMO) in *T. reesei* [2]. Their induction was mainly regulated by transcription factors (TFs), among which the regulator Xyr1 is considered as the master activator to induce the expression of genes encoding both cellulase and hemicellulase. Xyr1-A824V was verified to be the constitutively active mutant of Xyr1, which could significantly increase the expression of cellulase and hemicellulase, including the cellobiohydrolase-encoding genes (*cbh1* and *cbh2*) and the endo-β-1,4-xylanase-encoding genes (*xyn1* and *xyn2*) [3]. The repressor Cre1 mediates carbon catabolite repression (CCR) at presence of glucose, which results in the low secretion of cellulase and hemicellulase. A partial relief of CCR was found in a Cre1-deficient strain Rut-C30, which was used as an important industrial strain for the production of cellulase and hemicellulase [4]. Besides, Ace1 is another important repressor of cellulase and hemicellulase genes, because deletion of *ace1* resulted in an increase in the expression of all the main cellulase genes and major xylanase genes [5]. Other TFs including HAP2/3/5 complex, Ace2 and Rce1 also relate to regulation of the cellulase [2]. These TFs work synergistically to control the expression of lignocellulolytic enzymes. Particularly, engineering TFs could improve the production of cellulase [1].

Meanwhile, *T. reesei* is widely used for the production of recombinant enzymes [6]. For example, the lipase B of *Candida antarctica* and the cellobiose dehydrogenase of *Phanerochaete chrysosporium* could be produced by *T. reesei* [7, 8]. However, when the strong inducible promoters were used to dive the target enzyme production, the endogenous cellulases were also generated, causing the complex purification process. Thus, deletion of major cellulase genes was the efficient strategy adopted to increase the purity and yield of heterologous enzymes [9]. Nevertheless, multiple genetic manipulations need appropriate genetic tools and selection markers in *T. reesei*.

Traditional genetic approaches in *T. reesei* are relatively time-consuming, partly due to the low efficiency (about 5%) of homologous recombination (HR) [10]. The Cre/loxP system is another conventional technique for gene deletion, but it would leave scars in the genome during the Cre-mediated recombination [11]. I-SceI endonuclease-mediated system is a new powerful method; however, it needs the I-SceI restriction site inserted in advance at the target locus so that it is time-consuming and laborious [12]. In addition, the selection markers are limited in *T. reesei*, generally including *hph* (hygromycin) and *ptrA* (pyrithiamine), and *pyr4* (encoding an enzyme of the uridine biosynthesis pathway) [10, 13, 14]. Recently, the CRISPR/Cas9-mediated genome editing system derived from *Streptococcus pyogenes* has been established for gene editing in *T. reesei* [15].

During the development of CRISPR/Cas9 system, the expression of Cas9 and sgRNA (single guide RNA) is the major concern in *T. reesei*. In the first instance, when the Cas9-encoding gene *cas9* was integrated into the fungal genome for expression, it was found to be toxic to the fungal cells [15]. To avoid the integration of *cas9* into the genome, in vitro assembly of CRISPR/Cas9 ribonucleoproteins (RNPs), including the purified Cas9 protein and the in vitro transcribed sgRNAs, was applied to transient exposure of Cas9 in the cells, but this method is time-consuming [16]. Alternatively, the self-replicating plasmid-based method, where *cas9* was connected to the AMA1 replicator for extrachromosomal maintenance, was proved to be effortless for the Cas9 expression [13]. Preparation of multiple sgRNAs is another important problem for multi-gene editing. Initially, in vitro synthesis of multiple mature sgRNAs was adopted in *T. reesei*, but the stability of sgRNAs was an issue [15]. Similarly, when the RNPs were applied to genome editing in filamentous fungi *Aspergillus clavatus* and *A. oryzae*, the multiple sgRNAs were also synthesized in vitro [17]. Recently, in vivo expression of multiple sgRNAs was achieved using three strategies in fungi. The first strategy is to use the RNA polymerase III-based promoter *5S rRNA* for transcription of multiple gRNAs in *A. niger,* which was quite time-consuming due to construction of individual gRNA expression cassettes [18]. It was recently found that the endoribonuclease Csy4 from *Pseudomonas aeruginosa* could recognize a 28 ribonucleotides stem-loop sequence and cleave at position 20 to simultaneously produce multiple sgRNAs from one primary transcript, but Csy4 need to be expressed in the cells, resulting in additional manipulations [19]. Up to now, the most efficient strategy is using the tandemly arrayed tRNA−gRNA architecture, where an endogenous RNases could cleave the ends of tRNA precursors (pre-tRNAs) to generate multiple sgRNA transcripts from one primary transcript [20]. The architecture has been described in the filamentous fungus *Aspergilli* for expression of multiple sgRNAs [21]. There was no report on the tRNA-spacer-gRNA system adapted for *T. reesei.*

In this study, we developed an efficient CRISPR/Cas9 genome editing system based on a multiple sgRNA processing platform, which utilized the glycine tRNA (tRNA^Gly^) processing mechanism to generate multiple sgRNAs from a single transcript for genome editing in *T. reesei*. First, the tRNA−gRNA array-based multi-sgRNA processing platform was established. Then, *xyr1-A824V* was overexpressed at the *ace1* locus through homologous recombination mediated by the system for strain impovement of *T. reesei*. Furthermore, the system was applied to heterologously express glucose oxidase with the simultaneous deletion of *cbh1* and *cbh2* (two cellobiohydrolase coding genes) to exhibit the efficiency of the tRNA−gRNA array-based CRISPR−Cas9 editing system in *T. reesei.*

## Results and discussion

### Construction of the tRNA−gRNA array-based multi-sgRNA processing platform for efficient CRISPR−Cas9 gene editing in ***T. reesei***

**Fig. 1:**
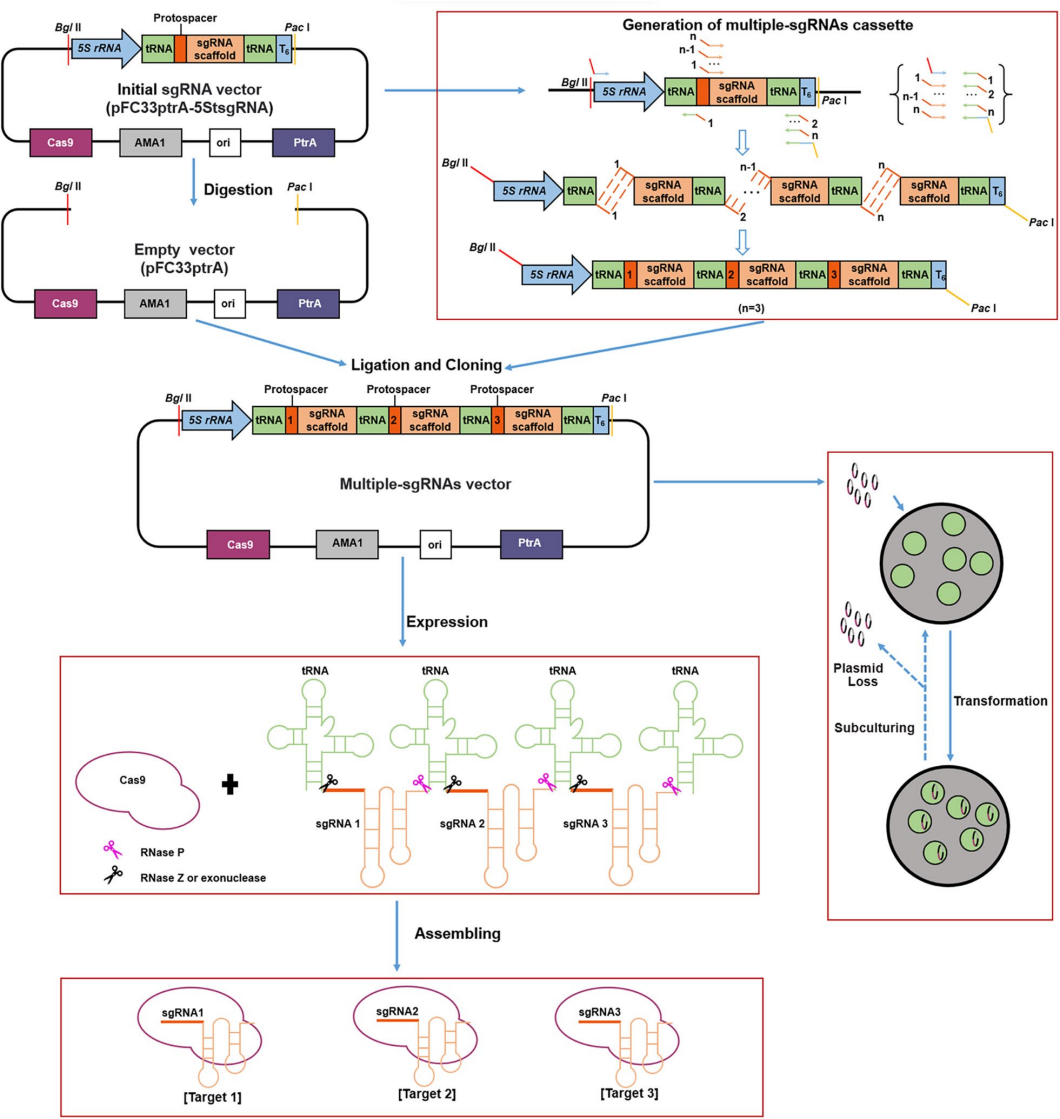
Graphical overview of the tRNA−gRNA array-based CRISPR/Cas9 system for multiplex genome editing in *T. reesei*. The initial sgRNA vector is composed of the fragments containing the Cas9 cassette, the tRNA−gRNA array cassette, the PtrA cassette (selection marker) and AMA1 (autonomously replicating sequence). The tRNA−gRNA array cassette located on the initial sgRNA vector carries the *5S rRNA* promoter, the tandemly arrayed tRNA−gRNA architecture (including tRNA, protospacer and sgRNA scaffold) and the T_6_ terminator. Then, the cassette serves as a template for PCR amplification to generate new cassettes that can carry multiple sgRNAs (the detailed PCR amplification process is described in Materials and Methods). The multi-sgRNA cassette is ligated into the empty vector to generate the multi-sgRNA expression vector. After transformed to *T. reesei*, the Cas9 endonucleases and the primary transcript of the tRNA−gRNA array cassette are expressed. Subsequently, the primary transcript is cleaved by endogenous RNase P, RNase Z and exonuclease at the 5ʹ and 3ʹ-ends of tRNA to release multiple functional sgRNAs. The excised functional sgRNAs and the Cas9 endonucleases are then assembled into the mature Cas9/sgRNA complex in vivo, which can target multiple sites simultaneously for multiplex genome editing. In addition, the AMA1-based plasmids can be lost after successive subculture, enabling the recycling of selection markers and multiple rounds of genetic engineering

The targeting capability of CRISPR/Cas9 system is commonly limited by the sgRNA-expressing device [22, 23]. Thus, the strategies of producing numerous sgRNAs from a single polycistronic gene have been developed, especially using synthetic genes with tandemly arrayed tRNA−gRNA architecture [20, 21]. The tRNA−gRNA array-based multi-sgRNA processing system utilized endogenous tRNA processing mechanism that the tRNA precursors could be identified and cleaved by RNases to generate multiple sgRNA transcripts from one primary transcript [20]. Although the CRISPR/Cas9 technology has been established in *T. reesei*, the convenient simultaneous multi-gene targeting method is still needed. Considering tRNA and its processing system are essentially conserved in all living organisms, we sought to build the tRNA-based multi-sgRNA processing platform to boost the CRISPR/Cas9 multiplex editing capability in *T. reesei*. The components of the tRNA−gRNA architecture for producing multi-sgRNA are shown in Fig. 1. The designed multi-sgRNA expression cassette contains the *5S rRNA* promoter, the tandemly arrayed tRNA−gRNA architecture (including tRNA, protospacer and sgRNA scaffold) and the T_6_ terminator. The glycine tRNA (tRNA^Gly^) was selected, because of its small size and efficient use in diverse organisms [20, 21, 24]. Here, the putative tRNA^Gly^ sequences were predicted and obtained by the BLASTn search from the genome sequences of *T. reesei* QM6a (http://genome.jgi-psf.org/Trire2/Trire2.home.html) (Additional file 1: Fig. S1B). The RNA Polymerase III promoter *5S rRNA* was selected to drive expression of a single sgRNA transcript containing a series of sgRNAs with tRNA^Gly^ as spacer [25]. All the elements for multi-sgRNA expression would be ligated into the plasmid pFC33ptrA (Fig. 1), an extra-chromosomal *cas9* expressing plasmid with *ptrA* as a selection marker, which is derived from the plasmid pFC330 that contains the self-replicating element AMA1 and the Cas9 expression cassette [26]. Generation of the multi-sgRNA cassette would be achieved using the Double-joint PCR strategy with the primers containing protospacer as overlap.

**Fig. 2:**
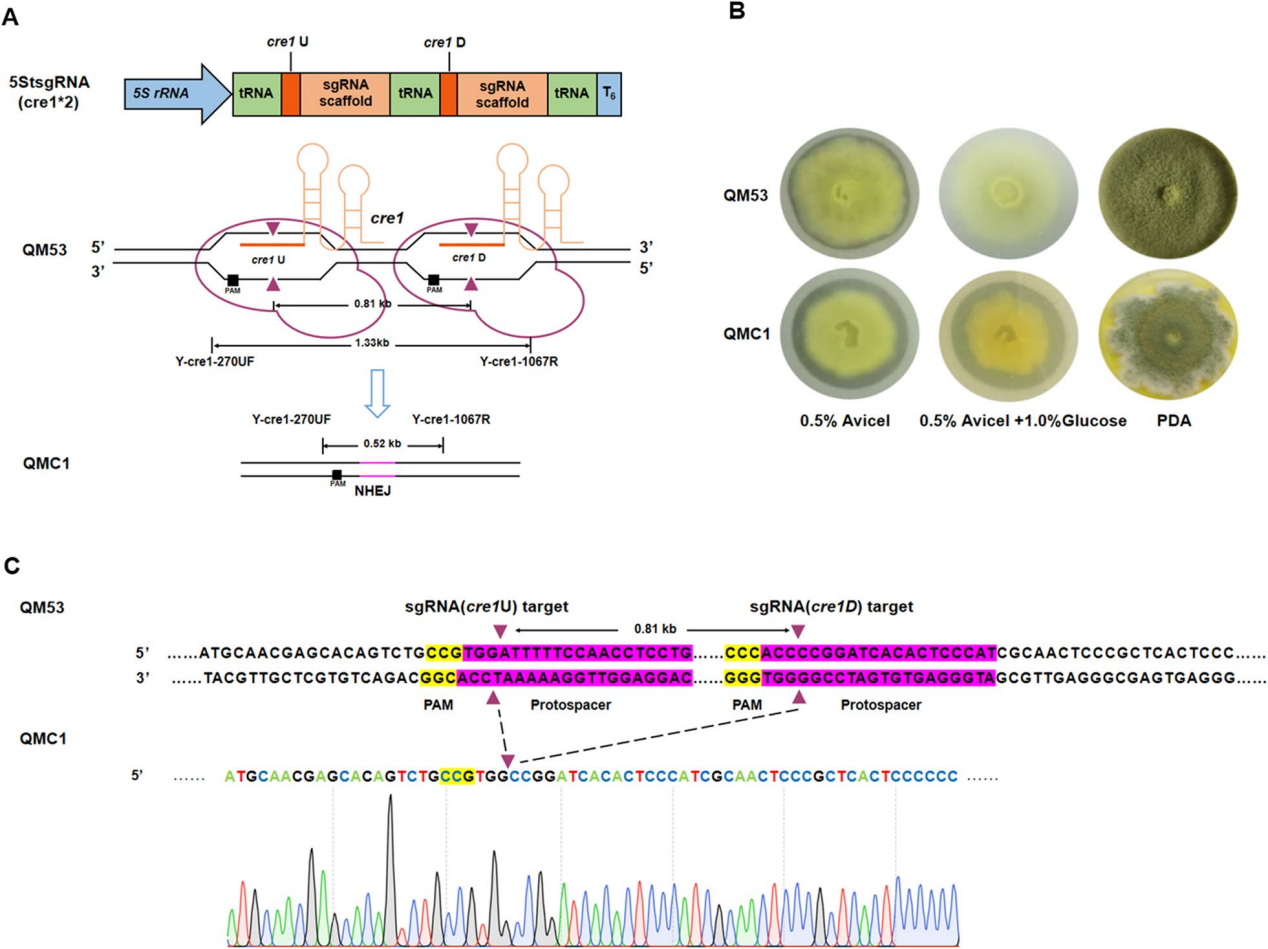
Deletion of the *cre1* gene in *T. reesei* by the tRNA−gRNA array-based multi-sgRNA processing platform. **A** Schematic diagram of the *cre1* gene deletion process in *T. reesei*. After transformed to the parental strain QM53, two sgRNAs (sgRNA-*cre1*U and sgRNA-*cre1*D) were co-expressed from the tRNA−sgRNA array cassette named as 5StsgRNA (cre1*2). Subsequently, the Cas9 endonucleases were recruited to the target sites of *cre1* by sgRNA-*cre1*U and sgRNA-*cre1*D to generate two double-strand breaks (DSBs, ▲ represents the sites for DSB) adjacent to the PAM sequence. After losing of the middle fragment of the two DSBs, the chromosome could be repaired by non-homologous end-joining (NHEJ) to complete deletion of *cre1*. The final Δ*cre1* strain was named as QMC1. **B** Growth of the Δ*cre1* strain QMC1 and the parental strain QM53 on the MM plate containing Avicel (0.5%) as the sole carbon source, the MM plate containing Avicel (0.5%) plus glucose (1.0%) as the mixed carbon sources and the PDA plate. **C** Sequence alignment of the *cre1* target locus in QMC1. The sequencing result of *cre1* from QMC1 was shown and the *cre1* sequence from the parental strain QM53 was listed as the wild-type reference gene (the two PAM sequences are highlighted in yellow and protospacer sequences in pink)

To test whether the tRNA^Gly^-based multi-sgRNA processing platform could be used to liberate different sgRNAs, an sgRNA expression cassette, named as 5StsgRNA(cre1*2), was constructed, which contained two sgRNAs targeting loci (*cre1*U and *cre1*D) of the *cre1* gene encoding the carbon catabolite repressor 1 (Cre1) (Fig. 2A). The cassette was ligated into the pFC33ptrA vector to yield the final plasmid pFC33ptrA-5StsgRNA(cre1*2) and then introduced into *T. reesei* QM53. The transformed protoplasts were screened on the MM plates containing pyrithiamine as the selection marker. PCR analysis showed that the transformants produced a 0.52 kb amplification product while the parental strain QM53 generated a 1.33 kb product with the primer pair Y-cre1-270UF/Y-cre1-1067R, suggesting a 0.81 kb fragment of *cre1* deleted in the transformant QMC1 (Additional file 1: Fig. S3A). In addition, an editing efficiency of 43.75% (7/16) was found using the two sgRNAs (Additional file 1: Fig. S3A: Table S5). Meanwhile, the secretion of cellulase by QMC1 was determined on the solid Avicel-supplemented medium. It was found that QMC1 showed a bigger cellulolytic halo than QM53, indicating the enhancement of cellulase secretion due to deletion of *cre1* (Fig. 2B). Furthermore, QMC1 was detected on the MM plate containing Avicel and glucose. It was discovered that QMC1 showed a cellulolytic halo while QM53 did not exhibit a cellulolytic halo, suggesting that carbon catabolite repression (CCR) was probably reduced due to deletion of *cre1* in QMC1 (Fig. 2B). However, compared with the parental strain QM53, QMC1 produced less spores and smaller colony (Fig. 2B). These features in QMC1 were consistent with characteristic of deletion of *cre1* [27]. In addition, the *cre1* locus in the genome of QMC1 was verified by sequencing, and the 0.81 kb-fragment deletion of *cre1* between the two sgRNAs (*cre1*U and *cre1*D) is displayed in Fig. 2C, which demonstrated that *cre1* was successfully deleted by the tRNA-based CRISPR−Cas9 system and the deletion locus was repaired by the non-homologous end-joining (NHEJ) pathway. These results indicated that tRNAs^Gly^ as the spacer could be processed to release two sgRNAs, which induced Cas9 to cut the targets in the genome of *T. reesei*. Generally, removal of 5’ leaders and 3’ trailers in the tRNA maturation processing is utilized to release sgRNAs [21]. It is well known that the 5’ end is removed by an endonuclease RNase P in all genetic systems [28]. However, the 3’ end is processed by the RNase Z or successively cleaved by RNase E and 3’ exonuclease in prokaryotes [29], while it is removed by RNase Z or exonuclease in eukaryotes [30]. Meanwhile, the increasing evidence suggests that 3’ exonucleases also participate in the removal of the 3’ trailer sequence in eukaryotes [30]. In addition, there are short and long forms of RNase Z in organisms, designated as RNase Z^S^ and RNase Z^L^, respectively, whereas RNase Z^L^ only occurs in eukaryotes [30]. Indeed, there is only one RNase Z^L^-encoding gene in the genome of *T. reesei* (Protein Id: 61701 in the genome of *T. reesei* QM6a (http://genome.jgi-psf.org/Trire2/Trire2.home.html). Moreover, several 3’ exonuclease-encoding genes are annotated in the genome of *T. reesei* (Protein Id: 47766; 52956; 42264; 103993; 112292 and 106797). Thus, the liberation of two sgRNAs to mediate deletion of *cre1* by the tRNA^Gly^-based multi-sgRNA processing platform was highly due to the available tRNA-processing enzymes in *T. reesei.*

### Homologous recombination mediated by the tRNA–gRNA array-based CRISPR–Cas9 editing system to express *xyr1* at the* ace1* locus in *T. reesei*

**Fig. 3:**
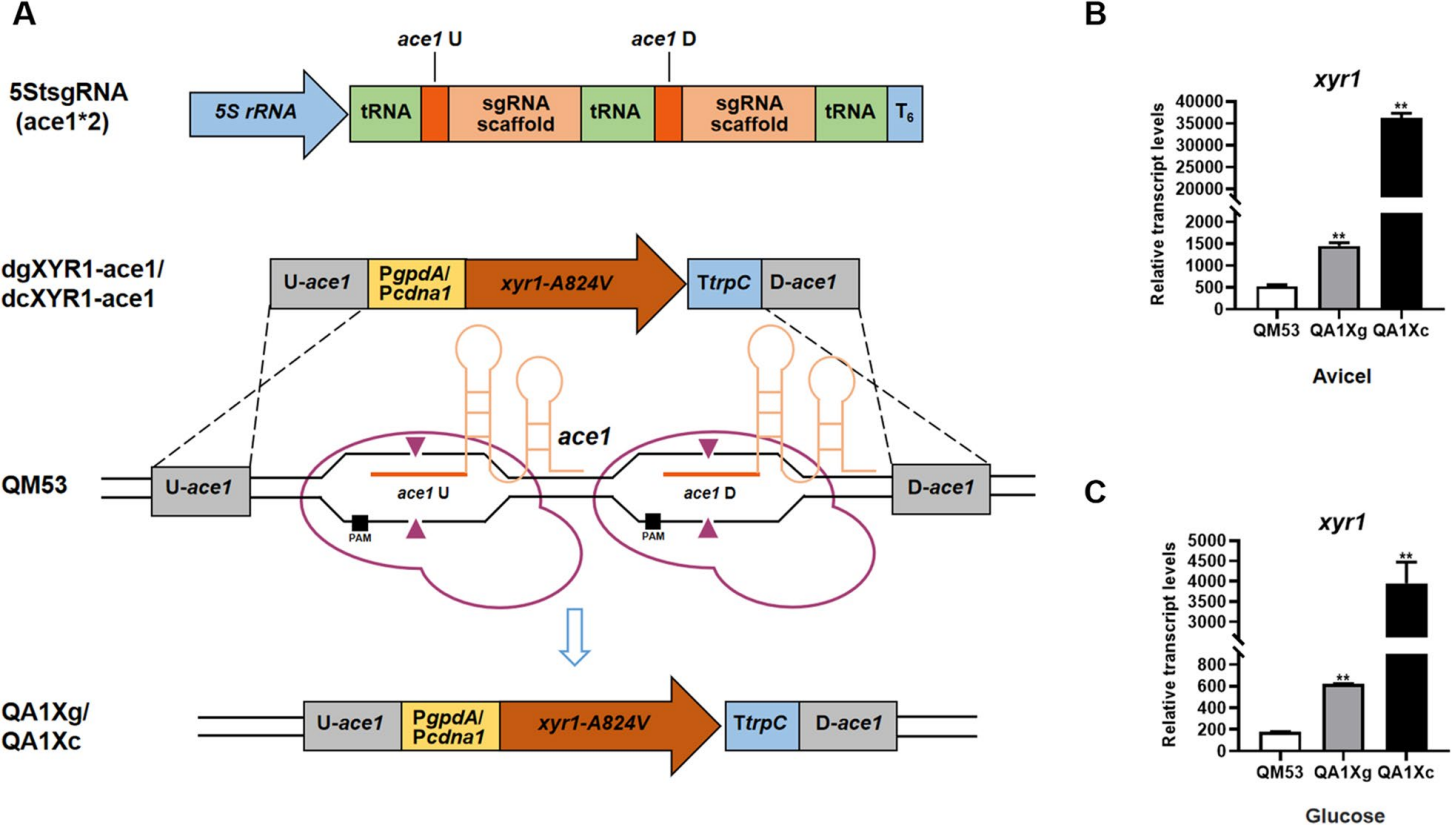
Overexpression of the *xyr1-A824V* gene at the *ace1* locus in the *T. reesei* genome through homologous recombination mediated by the tRNA−gRNA array-based CRISPR−Cas9 editing system. **A** Schematic diagram of the *xyr1-A824V* overexpression process. After transformed to the parental strain QM53, two sgRNAs (sgRNA-*ace1*U and sgRNA-*ace1*D) were co-expressed from the tRNA−gRNA array cassette named as 5StsgRNA (ace1*2). Subsequently, Cas9 endonucleases were recruited to the target sites of *ace1* by sgRNA-*ace1*U and sgRNA-*ace1*D to generate two double-strand breaks (DSBs, ▲). The broken chromosome can be repaired by homologous recombination (HR) pathway using donor DNA (the *xyr1-A824V* expression cassette)*.* The cassette named as dgXYR1-ace1 contains the *gpdA* promoter (P*gpdA*), the gene coding region of *xyr1-A824V*, the *trpC* terminator T*trpC*, the upstream homologous arm of *ace1* (U-*ace1*) and the downstream homologous arm of *ace1* (D-*ace1*). In addition, the cassette named as dcXYR1-ace1 has only the *cdna1* promoter P*cdna1* that differs from dgXYR1-ace1. The final *xyr1-A824V* overexpressing strains constructed with these two cassettes were named as QA1Xg and QA1Xc respectively. **B**, **C** Transcript levels of *xyr1* in the *xyr1-A824V* overexpressing strains QA1Xg, QA1Xc and the parental strain QM53 at 72 h under Avicel culture conditions and glucose culture conditions, respectively. Data are the Mean ± SD of the results from three independent experiments. Significant differences were analyzed using *t* test. (*p < 0.05, **p < 0.01.)

In the CRISPR–Cas9 system, sgRNAs can identify and bind the 20 nucleotide sequences, named as protospacer, upstream of the protospacer adjacent motif (PAM) NGG site to induce the Cas9–sgRNA complex to the target DNA, and then Cas9 participates in shearing 3–4 bp upstream of PAM to form a DNA double-strand break (DSB) [1]. Subsequently, the DSB will be repaired by NHEJ as the dominant repair pathway, or through the homologous recombination (HR) pathway with the help of exogenous donor fragments to exchange or integrate gene sequences [31]. Naturally occurring HR relies on a strand break that generates coincidentally at the target locus, whereas the efficiency of HR could be significantly enhanced by the DSB generated by the Cas9–sgRNA complex near the target locus [22, 32]. To verify whether the CRISPR–Cas9 editing system could operate via HR on the target gene loci in *T. reesei*, two *xyr1-A824V* overexpression cassettes under the constitutive promoters P*gpdA* (the gene encodes glyceraldehyde-3-phosphate dehydrogenase from *A. nidulans*) and P*cdna1* (the gene encodes the hypothetical protein Trire2:110,879 from *T. reesei*) targeting the *ace1* locus were constructed as two donor DNAs (dDNAs), named as dgXYR1-ace1 and dcXYR1-ace1, respectively. Then the two overexpression cassettes were transformed into the protoplasts of *T. reesei* QM53 together with the plamid pFC33ptrA-5StsgRNA(ace1*2) containing two sgRNAs targeting *ace1* designed near the homologous arms, which could facilitate creating two DSBs with the help of Cas9 to improve the HR frequency. The resultant strains were named QA1Xg and QA1Xc, respectively (Fig. 3). After screening on the plates added with pyrithiamine, *T. reesei* colonies were verified by PCR. It was found that 60% (9/15) of the transformants contained the donor cassettes integrated at the *ace1* locus (Additional file 1: Fig. S3B, Table S5). In the control experiment, the HR frequency was only 40% (6/15), where the donor dgXYR1-ace1 and the plasmid pFC33ptrA-5StsgRNA(ace1) containing one sgRNA targeting *ace1* were co-transformed into the protoplasts of *T. reesei* QM53 (Date not shown)*.* Thus, the tRNA–gRNA array-based CRISPR–Cas9 system exhibited higher HR efficiency than that of the naturally occurring HR, which was reported to be only 5% [10], and the efficiency could be further increased when using two sgRNAs in comparison with the single gRNA.

Subsequently, the transcript levels of *xyr1* in QA1Xg and QA1Xc were confirmed by RT-qPCR (Fig. 3B, C). When the strains were cultivated in the liquid medium with Avicel or glucose as the carbon source, the transcript levels of *xyr1* in QA1Xg and QA1Xc were higher than that in QM53, demonstrating that *xyr1-A824V* was overexpressed (Fig. 3B, C). Specifically, the transcript levels of *xyr1* in QA1Xg and QA1Xc were 1.73- and 67.24-fold higher than that of the parental strain QM53 with Avicel as the carbon source, respectively, and the transcript levels of *xyr1* QA1Xg and QA1Xc were 2.50- and 21.29-fold higher than that of QM53 using glucose as the carbon source (Fig. 3B, C). It was found that the transcript level of *xyr1* in QA1Xc was much higher than QA1Xg. This is because the promoter strength of *cdna1* was higher than *gpdA* [33]. Meanwhile, the transcript levels of *xyr1* in all strains were higher in Avicel than in glucose (Fig. 3B, C). It was reported that the Xyr1 expression was negatively regulated by the CRE1-dependent CCR and the repressor ACE1 [34]. Considering that *ace1* was replaced by *xyr1-A824V* in this study, this phenomenon that *xyr1* was regulated to lower level in glucose than in Avicel could be ascribed to the Cre1-mediated CCR. Therefore, expression of *xyr1* at the *ace1* locus was successfully achieved by homologous recombination, which was efficiently mediated through the tRNA–gRNA array-based CRISPR–Cas9 editing system in *T. reesei*. In addition, QA1Xg was cultured on the non-selective medium (the MM plate without pyrithiamine) for two generations, and the sequences including *ptrA,* sgRNA or *Cas9* could not be amplified, indicating the loss of the pFC33ptrA-5StsgRNA(ace1*2) plasmid in *T. reesei* (Additional file 1: Fig. S4).

### Overexpression of *xyr1-A824V *at the *ace1* locus led to the increased production of cellulase and xylanase

*T. reesei* produces different lignocellulolytic enzymes for degradation of lignocellulosic biomass, including cellulase and hemicellulase [1]. Nonetheless, the lignocellulase system was proven to be present in suboptimal ratio for efficient deconstruction of lignocellulosic substrates [2]. Thus, genetic engineering of *T. reesei* strains has been applied to optimize the components of cellulase and hemicellulase to improve the hydrolysis performance [2]. It is known that the production of lignocellulolytic enzymes is regulated by different transcriptional factors, including activators and repressors [1]. Xyr1-A824V is a constitutively active mutant of Xyr1, the major activator of lignocellulase, while Ace1 is a well-known repressor [3, 5]. Efficient production of cellulase and hemicellulase in *T. reesei* could be achieved by the combined manipulation of both activators and repressors [2]. For example, the constitutive expression of *xyr1* could produce cellulase and xylanase on glucose due to relieving from the carbon catabolite repression in *T. reesei* [34, 35]. Here, the effect of the *xyr1-A824V* overexpression at the *ace1* locus (deletion of *ace1*) on the production of cellulase and xylanase was investigated.

**Fig. 4:**
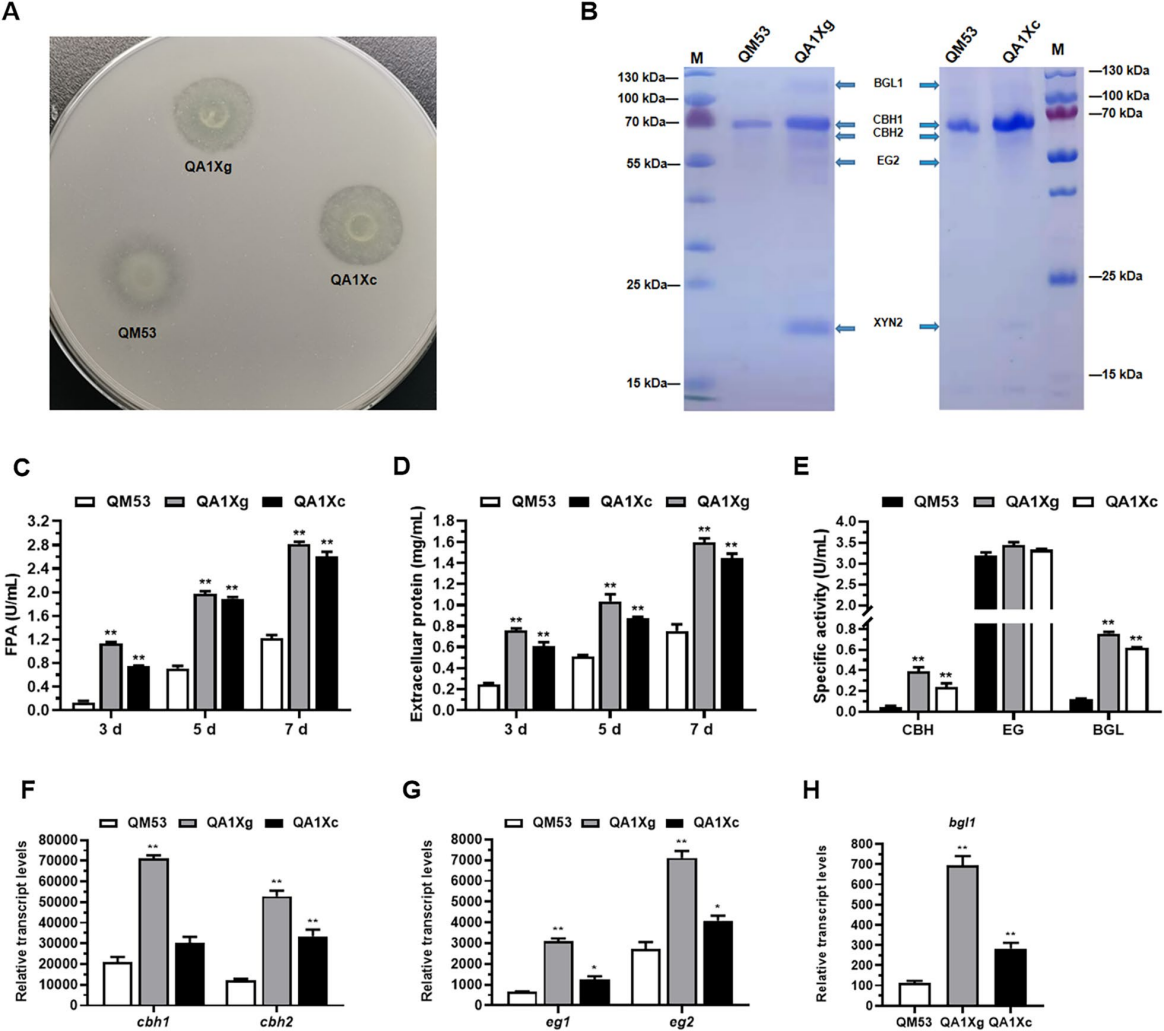
Cellulase production in the *xyr1-A824V* overexpressing strains QA1Xg and QA1Xc under Avicel culture conditions. **A** Qualitative evaluation of the ability to secrete cellulase using the MM plate containing 0.5% ball-milled Avicel as sole carbon source. **B** SDS-PAGE analysis of the fermentation supernatants from QA1Xg, QA1Xc and the parental strain QM53 under Avicel culture conditions. **C** The activities of FPA and **D** the amount of extracellular protein at 3 days, 5 days, and 7 days under Avicel culture conditions, respectively. **E** The activities of CBH, EG and BGL at 7 days under Avicel culture conditions. **F** The transcript levels of the cellobiohydrolase genes *cbh1* and *cbh2.* **G** The transcript levels of the endoglucanase genes *eg1* and *eg2.* **H** The transcript level of the β-glucosidase gene *bgl1*. All values were normalized to *actin* expression under same conditions. Data are the Mean ± SD of the results from three independent experiments. Significant differences were analyzed using* t* test. (*p < 0.05, **p < 0.01.)

**Fig. 5:**
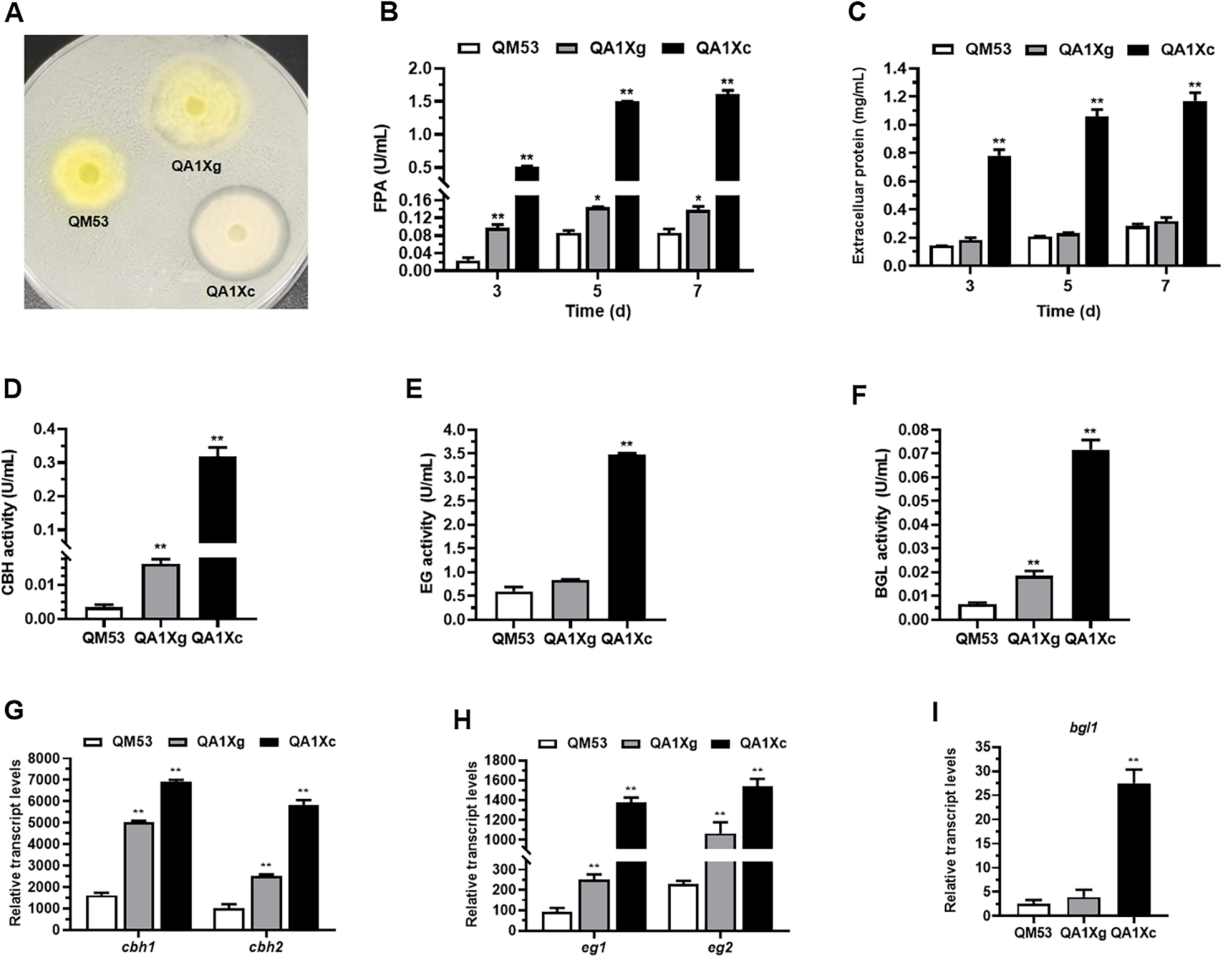
Cellulase production in the *xyr1-A824V* overexpressing strains QA1Xg and QA1Xc under glucose culture conditions. **A** Qualitative evaluation for the ability to secrete cellulase using the MM plate containing both 0.5% ball-milled Avicel and 1% glucose as carbon sources. **B** The activities of FPA and **C** the amount of extracellular protein at 3 days, 5 days, and 7 days of glucose culture conditions. **D** CBH activity, **E** EG activity and **F** BGL activity at 7 days under glucose culture conditions. **G** The transcript levels of the cellobiohydrolase genes *cbh1* and *cbh2* at 72 h*.* **H** The transcript levels of the endoglucanase genes *eg1* and *eg2* at 72 h*.* **I** The transcript level of the β-glucosidase gene *bgl1* at 72 h. All values were normalized to *actin* expression under same conditions. Data are the Mean ± SD of the results from three independent experiments. Significant differences were analyzed using *t* test. (*p < 0.05, **p < 0.01)

First, the secretion of cellulase by QA1Xg and QA1Xc was assessed on the MM plates containing Avicel as the carbon source (Fig. 4A). It was found that QA1Xg and QA1Xc presented larger cellulolytic halos around the colonies than the parental strain QM53, indicating that overexpression of *xyr1-A824V* at the *ace1* locus promoted secretion of cellulase. Furthermore, the strains were cultivated in the liquid medium containing Avicel as the sole carbon source to detect the cellulase production. The fermentation supernatant at 7 days was used for analysis. SDS-PAGE showed that the bands of CBH, BGL and endo-β-1,4-xylanase 2 (XYN2) were more intense in QA1Xg and QA1Xc than those of QM53, indicating the higher CBH, BGL and XYN2 production in QA1Xg and QA1Xc (Fig. 4B). The whole cellulase production represented by filter paper activity (FPA) in QA1Xg and QA1Xc achieved 2.82 U/mL and 2.60 U/mL, respectively, which was elevated 1.33- and 1.14-fold compared to that of the QM53 (1.21 U/mL) (Fig. 4C). As shown in Fig. 4D, the production of extracellular proteins in QA1Xg and QA1Xc reached 1.59 mg/mL and 1.44 mg/mL on day 7, respectively, which was 1.12- and 0.92-fold higher than that in QM53 (0.75 mg/mL). Furthermore, the activities of CBH, BGL and EG in QA1Xg achieved 0.39 U/mL, 0.75 U/mL and 3.44 U/mL, respectively, which were 7.07-, 5.41- and 0.07-fold than those of QM53 (Fig. 4E). In addition, the activities of CBH, BGL and EG in QA1Xc reached 0.23 U/mL, 0.62 U/mL and 3.33 U/mL, respectively, which were 3.76-, 4.30- and 0.04-fold higher than those in QM53 (Fig. 4E). Meanwhile, transcription of the cellulase genes (*cbh1*, *cbh2*, *egl1*, *egl2* and *bgl1*) was also monitored by RT-qPCR. It was found that the transcript levels of all cellulase genes were significantly upregulated in QA1Xg and QA1Xc compared to those of QM53 (Fig. 4F–H). Meanwhile, the transcript levels of all cellulase genes in QA1Xg were higher than QA1Xc (Fig. 4F–H). As shown above, the *xyr1-A824V* overexpression at the *ace1* locus enhanced production of cellulase using Avicel as the carbon source. Nonetheless, we also found that although the transcript level of *xyr1* in QA1Xc was higher than QA1Xg, the cellulase production of QA1Xc was slightly lower than QA1Xg. In other words, the production of cellulase did not tally with the transcript level of *xyr1-A824V*, which was in accordance with the results reported by Shen et al. (2022), where the P*egl2*-driven *xyr1* overexpression strain displayed highest cellulase production with relatively lower transcript abundance of *xyr1* than the two strong promoters P*cbh1* and P*cdna1* [36]. It is reported that Xyr1 must experience posttranslational modifications to convert into its active form, so it may occur at a high concentration that is in an inactive form due to lacking an essential modifications [34]. In addition, the excessive mRNA of *xyr1* may also induce translation suppression or degradation by post-transcriptional gene silencing (PTGS) [36]. Thus, it could be speculated that a large amount of *xyr1-A824V* may be in an inactive form or post-transcriptionally degraded in QA1Xc. Subsequently, the secretion of cellulase by QA1Xg and QA1Xc was detected on the Avicel and glucose-supplemented plates (Fig. 5A). QA1Xc and QA1Xg showed intense cellulolytic halos around the colonies, while no cellulolytic halo was observed for the parental strain QM53, suggesting overexpression of *xyr1-A824V* at the *ace1* locus alleviated CCR on the glucose.

Then, the strains were grown in shake flasks using glucose as the sole carbon source to detect the cellulase production. The activities of FPA, CBH, BGL and EG were 1.61 U/mL, 0.32 U/mL, 0.07 U/mL, and 3.47 U/mL, respectively, in QA1Xc on day 7, which were 10.65-, 18.88-, 2.80-, and 3.15-fold higher than those of QA1Xg, while the cellulase activities were barely detectable in QM53, which was due to the well-known CCR repressed by glucose in a Cre1-dependent manner (Fig. 5B, D, E and F). QA1Xc exhibited a protein concentration of 1.17 mg/mL, representing approximately 2.77-fold higher than QA1Xg (0.31 mg/mL) and 3.15-fold higher than QM53 (Fig. 5C). Furthermore, RT-qPCR was used to quantify the transcript levels of cellulase genes. It was discovered that the transcript levels of all cellulase genes were upregulated in QA1Xc and QA1Xg than those in QM53 (Fig. 5G–I), because these genes were directly repressed by glucose in the manner dependent on the carbon catabolite repressor Cre1 [2]. In contrast, the transcript levels of all cellulase genes in QA1Xc were higher than those in QA1Xg. These findings are consistent with the observation of Lv et al. (2015), where overexpression of *xyr1* under the control of a copper responsive promoter *tcu1* relieved CCR to increase celluase production while the decreased expression of *xyr1* inhibited the cellulase production [35]. These results demonstrated that the constitutive expression of *xyr1-A824V* could release CCR to enhance cellulase production on glucose and further confirmed the importance of *xyr1-A824V* on strain improvement for cellulase production.

**Fig. 6:**
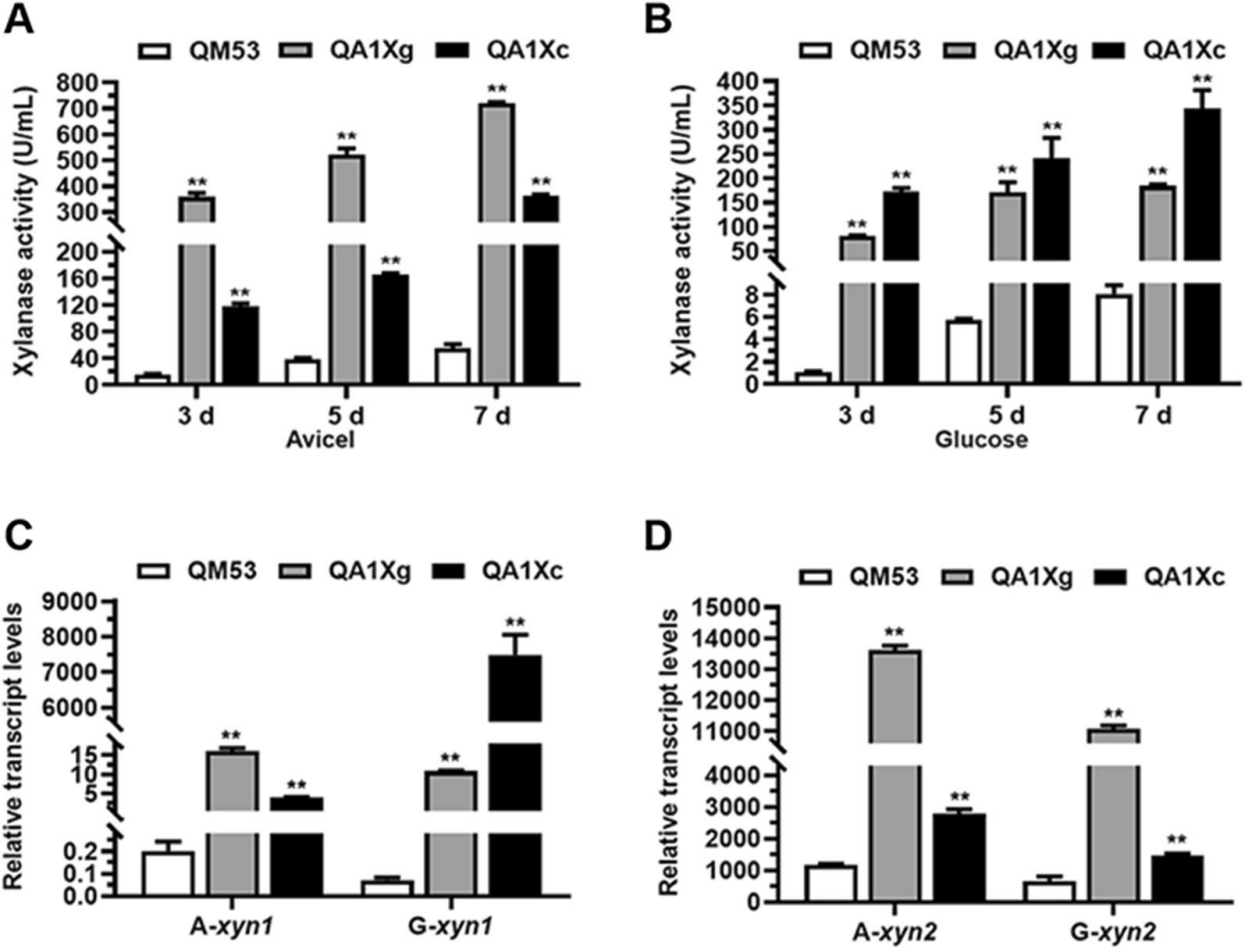
Xylanase production in the *xyr1-A824V* overexpressing strains QA1Xg and QA1Xc under Avicel and glucose culture conditions. The xylanase activities at 3 days, 5 days, and 7 days under Avicel **A** and glucose (**B**) culture conditions. **C** The transcript levels of the major xylanase genes *xyn1* **C** and *xyn2* (**D**) at 72 h under Avicel and glucose conditions, respectively. All values were normalized to *actin* expression under same conditions. Data are the Mean ± SD of the results from three independent experiments. Significant differences were analyzed using *t* test. (*p < 0.05, **p < 0.01.)

To evaluate the effect of the *xyr1*-A824V overexpression at the *ace1* locus (deletion of *ace1*) on the production of xylanase, the strains QA1Xg and QA1Xc were cultivated using two different carbon sources: Avicel and glucose, respectively. The fermentation supernatants were used to detect xylanase production. Meanwhile, the transcript levels of the xylanase genes (*xyn1*, *xyn2*, *xyn3*, *xyn4* and *xyn5*) were investigated. When Avicel was used as the carbon source, the xylanase activities of QA1Xg and QA1Xc on day 7 were 721.39 U/mL and 363.77 U/mL, which were 11.98- and 5.54-fold higher than that of the parental strain QM53 (55.55 U/mL), respectively (Fig. 6A). Meanwhile, QA1Xg and QA1Xc both had a considerably higher transcript levels of the xylanolytic genes (*xyn1, xyn2, xyn3*, *xyn4* and *xyn5*) than QM53 (Fig. 6C, D and Additional file 1: Fig. S2). In addition, the transcript levels of the xylanolytic genes in QA1Xg were higher than those in QA1Xc (Fig. 6C, D and Additional file 1: Fig. S2). These results demonstrated that overexpression of *xyr1-A824V* at the *ace1* locus also enhanced xylanase activity on Avicel. On glucose, the xylanase activities of QA1Xg and QA1Xc on day 7 were 184.40 U/mL and 343.81 U/mL, which were 21.87- and 41.65-fold higher than that of QM53 (8.06 U/mL), respectively (Fig. 6B). Meanwhile the transcript levels of the xylanolytic genes in QA1Xg and QA1Xc were also higher than those in QM53 (Fig. 6C, D and Additional file 1: Fig. S2). In addition, the transcript levels of most xylanase genes (*xyn1*, *xyn4* and *xyn5*) in QA1Xc were significantly increased compared to those in QA1Xg (Fig. 6C, Additional file 1: Fig. S2B and C). The relatively low xylanase expression in QM53 was probably due to the CCR mediated by Cre1 on glucose [37]. Exceptionally, the transcript levels of *xyn2* and *xyn3* in QA1Xc was lower than in QA1Xg (Fig. 6D and Additional file 1: Fig. S2A). Possible explanation might be that the glucose repression of the *xyn2/xyn3* expression presumably was not directly related to the Xyr1-relieved CCR [38, 39]. These results suggested that the overexpression of *xyr1-A824V* at the *ace1* locus also enhanced xylanase activity on glucose by releasing the CCR.

In previous reports, overexpression of *xyr1-V821F*/*xyr1* with the constitutive promoter P*pdc* at the *ace1* locus or by the RNAi-mediated silencing of *ace1* both resulted in improvement of cellulase and xylanase production in the Cre1-deficient mutant strain RUT-C30 [13, 40]. However, the effect of relief of CCR on glucose could not be explored in the Cre1-deficient strain. In addition, expression strength of *xyr1* was directly related to the level of enzyme production [36]. In this study, two promoters with different strengths were used to drive the expression of *xyr1-A824V* at the *ace1* locus in the Cre1-containing strain QM53, which was achieved by the tRNA–gRNA array-based CRISPR–Cas9 system developed in *T. reesei*. P*cdna1*-driven overexpression of *xyr1-A824V* resulted in higher enzyme production on glucose than P*gpdA* (Figs. 5 and ). Especially, more xylanase was produced on glucose in the Cre1-containing strain QA1Xc (over 300 U/mL) than those in the Cre1-deficient strains PXAi20 and Br_TrR03 (below 120 U/mL), which were derived from *T. reesei* RUT-C30 [13, 40]. The reason was likely due to the role of Cre1 or the expression strength of Xyr1 in QA1Xc. What’ more, the tRNA–gRNA array-based CRISPR–Cas9 system developed here can express multiple sgRNAs to perform multi-gene manipulations in a single experiment. It was reported that this type of system was used for just two-round deletions of eight genes in *Saccharomyces cerevisiae*, resulting in a 30-fold increase in free fatty acid production [24]. In summary, the novel tRNA–gRNA array-based CRISPR–Cas9 system developed in *T. reesei* was not only used to increase the cellulase and xylanase production by overexpression of *xyr1-A824V* at the *ace1* locus but would edit more genes/pathways for strain improvement to construct hyper-cellulase/xylanase producers.

### Application of the tRNA–gRNA array-based CRISPR–Cas9 system to delete two endogenous genes (*cbh1* and *cbh2*) and express the heterologous gene *gox *in *T. reesei*

**Fig. 7:**
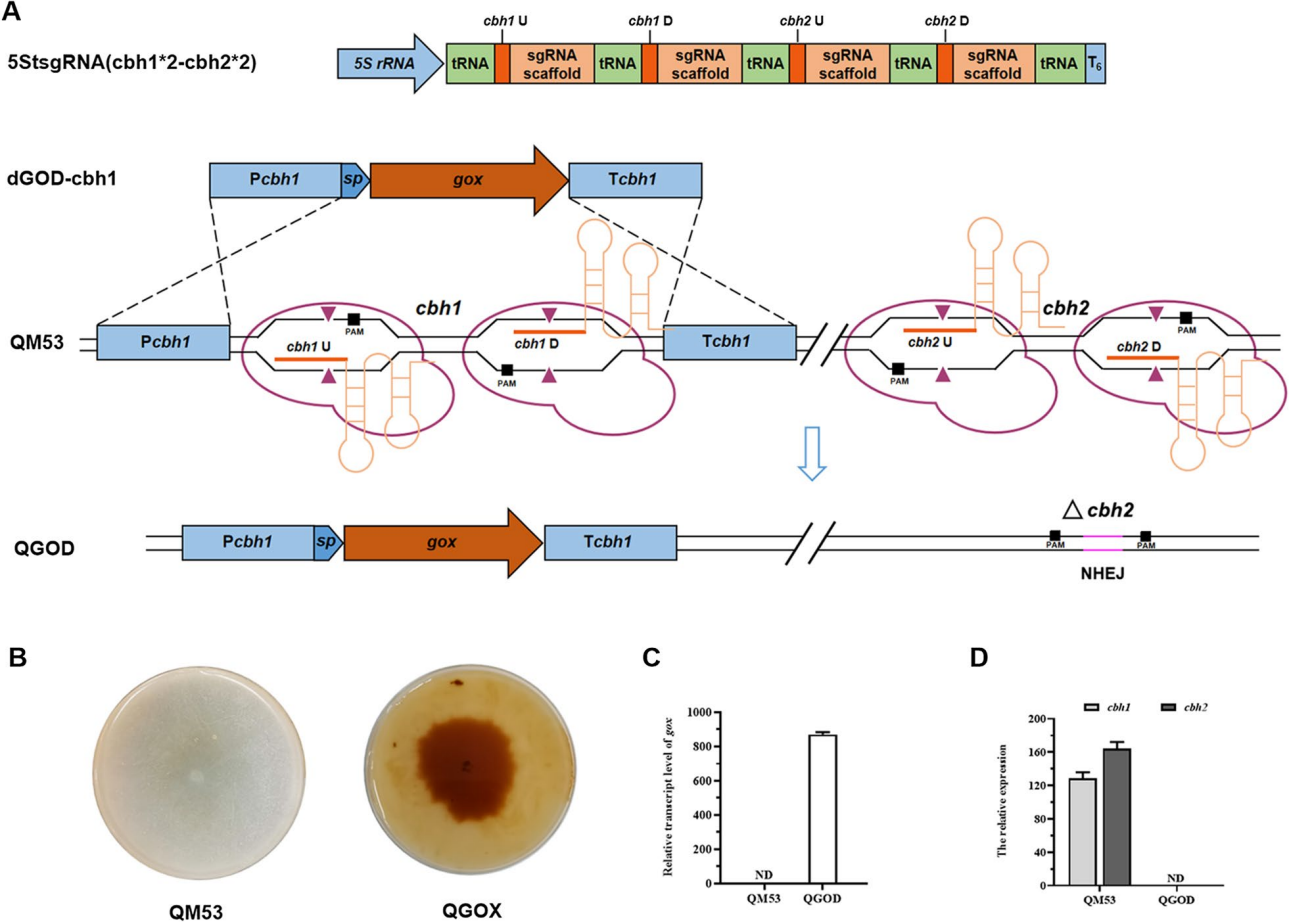
Simultaneous expression of the *gox* gene at the *cbh1* locus and deletion of the *cbh2* gene in the *T. reesei* genome by the tRNA−gRNA array-based CRISPR−Cas9 system. **A** Schematic diagram of the simultaneous multi-gene editing process. After transformed to the parental strain QM53, four sgRNAs (sgRNA-*cbh1*U, sgRNA-*cbh1*D, sgRNA-*cbh2*U and sgRNA-*cbh2*D) were co-expressed from the tRNA-sgRNA array cassette named as 5StsgRNA (cbh1*2-cbh2*2). Subsequently, Cas9 endonucleases were recruited to target sites of the *cbh1* and *cbh2* gene by sgRNA-*cbh1*U, sgRNA-*cbh1*D, sgRNA-*cbh2*U and sgRNA-*cbh2*D, generating double-strand breaks (DSB, ▲). The broken *cbh1* gene could be repaired by homologous recombination (HR) pathway using the donor DNA (the *gox* expression cassette)*.* The dDNA named as dGOD-cbh1 contains the *cbh1* promoter P*cbh1*, the CBH1 signal peptide (sp)*,* the gene coding region of *gox* and the *cbh1* terminator T*cbh1*. In addition, the broken *cbh2* gene could be repaired by non-homologous end-joining (NHEJ) to complete the deletion process. The final strain was named as QGOD. **B** Qualitative evaluation for the ability to secrete glucose oxidase using the plates where lactose was served as the sole carbon source and dye liquor was dumped containing o-dianisidine, HRP and glucose. **C** The transcript level of the *gox* gene in the strains QGOD and QM53. **D** The transcript levels of *cbh1* and *cbh2* in the strains QGOD and QM53. All values were normalized to *actin* expression under same conditions. Data are the Mean ± SD of the results from three independent experiments. ND = No Detection

*T. reesei* is expected to be an efficient microbial cell factory for the industrial production of recombinant proteins because of its powerful ability to secrete proteins. However, when using robust cellulase promoters to drive heterologous protein expression, a large number of endogenous cellulases also are secreted as byproducts and may induce the ER stress leading to the ER-associated protein degradation (ERAD) to degrade unfolded proteins [7, 41]. Since the cellobiohydrolases, CBH1 and CBH2, account for over 70% of the total secreted proteins in *T. reesei* [42], the gene loci of the responding genes, *cbh1* and *cbh2*, were selected as the Cas9–sgRNA targets to be deleted to reduce the background proteins and secretion pressure in this study*.* Meanwhile, the glucose oxidase-encoding gene *gox* from *A. niger* ATCC 9029 was heterologously expressed at the *cbh1* locus. A novel plasmid pFC33ptrA-5StsgRNA(cbh1*2-cbh2*2) containing four sgRNAs targeting different loci of *cbh1* (cbh1U and cbh1D) and *cbh2* (cbh2U and cbh2D) was constructed and transformed into *T. reesei* QM53 together with a donor DNA(dDNA) dGOD-cbh1, which was the glucose oxidase expression cassette driven by the promoter P*cbh1* and the terminator T*cbh1* of *cbh1* (Fig. 7A). After screening on the plates added with pyrithiamine, putative transformants were verified by PCR. It is known that glucose oxidase catalyzes the oxidation of β-D-glucose to D-glucono-δ-lactone and H_2_O_2_, which can oxidize o-dianisidine hydrochloride to a brown compound horseradish peroxidase (HRP) [43]. Thus, the strains that secreted glucose oxidase could be selected due to their colonies surrounded by brown halos on the dye liquor including o-dianisidine, HRP and glucose. Here, one of the putative transformants, namely QGOD, was tested on the lactose-supplemented plate containing the dye liquor. It was found that the colony of QGOD showed a brown halo while the colony of the parental strain QM53 could not produce brown halo, indicating the glucose oxidase secreted extracellularly by QGOD (Fig. 7B). Then the strain QGOD was fermented in the liquid media using lactose as the carbon source for glucose oxidase production. RT-qPCR analysis showed that the *gox* gene was transcribed in QGOD, whereas not detected in QM53 (Fig. 7C). Meanwhile, the transcripts of *cbh1* and *cbh2* were not detected in QGOD but found in QM53 (Fig. 7D). These results demonstrated that the *cbh1/cbh2* genes were deleted and meanwhile the *gox* gene was expressed at the *cbh1* locus in QGOD (Additional file 1: Fig. S3), indicating the simultaneous occurrence of several homologous recombination events at different loci in a single experiment. As for the efficiency of simultaneous manipulation of multiple genes in this study, 60.00% (12 of 20) of the transformants contained deletions of both *cbh1* and *cbh2* in the genome (Additional file 1: Fig. S3C, Table S5). It was reported that the frequency of simultaneous targeting events could be improved from 16 to 45% after optimization of the concentrations of gRNA and dDNA when using the in vitro transcribed sgRNA method [15]. Although the in vitro assembled RNPs could achieve the efficiency of 40.0% for two genome editing events with short homology arms in *A. clavatus*, it was laborious with adding chemical reagents and the selection marker was remained in the genome [17]. Moreover, the individual *cbh1/cbh2* deletion strain and the individual *gox* overexpression (at the *cbh1* locus) strain were also acquired (Additional file 1: Fig. S3C), suggesting that the multiple genotyped strains could be obtained in a single genome editing experiment in this study. Taken together, these results demonstrated that the tRNA–gRNA array-based CRISPR–Cas9 system developed here could be used to achieve multiplex genome editing by liberating sgRNAs, providing an efficient and convenient multi-gene editing tool in *T. reesei*.

### Overexpression of *gox* and deletion of *cbh1/cbh2* promoted production of glucose oxidase

**Fig. 8:**
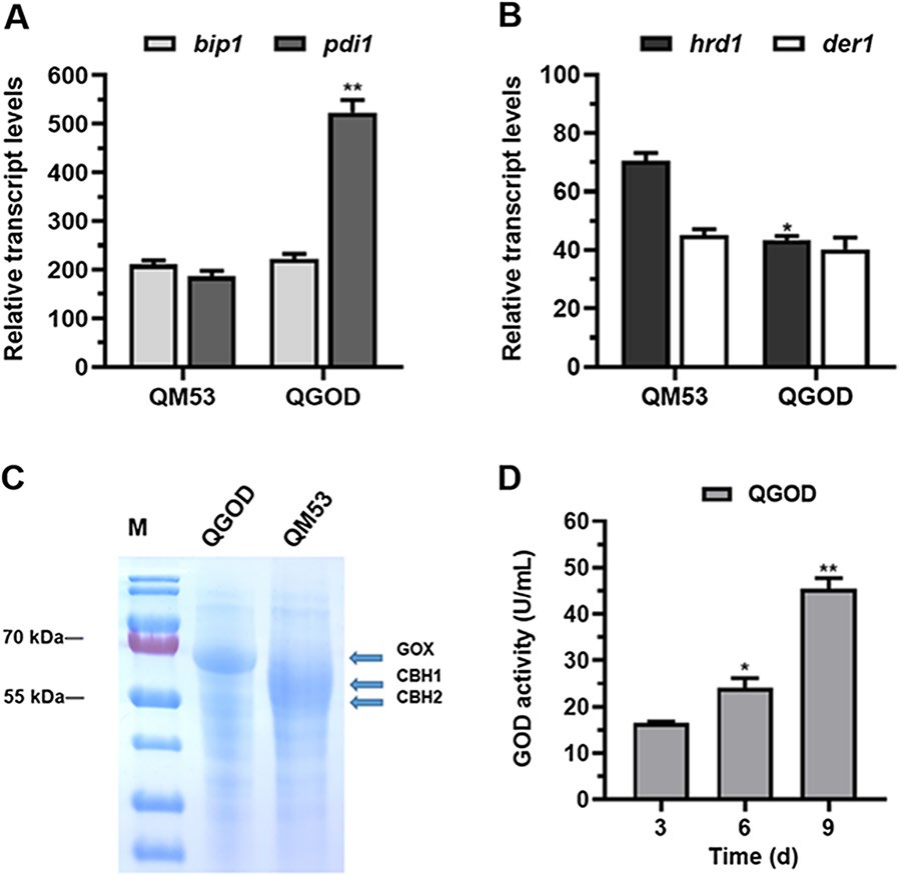
Production of glucose oxidase in the *gox* expressing strain QGOD. **A** The transcript levels of the UPR-related genes *bip1* and *pdi1* in QGOD and the parental strain QM53. **B** The transcript levels of the ERAD related genes *hrd1* and *der1* in QGOD and QM53*.* **C** SDS-PAGE analysis of the fermentation supernatants from QGOD and QM53 under lactose culture conditions. **D** The activities of glucose oxidase produced by QGOD at 3 days, 6 days and 9 days under lactose culture conditions. All values were normalized to *actin* expression under same conditions. Data are the Mean ± SD of the results from three independent experiments. Significant differences were analyzed using* t* test. (*p < 0.05, **p < 0.01.)

Heterologous proteins are likely to encounter problems such as improperly folding during secretion [2]. For example, the unfolded protein response (UPR) and ERAD were triggered in the heterologous laccase expression strain [41]. Thus, to detect whether the ER stress was induced by overexpression of the heterologous GOX in the QGOD, RT-qPCR was used to quantify the transcript levels of the genes related to the UPR (*bip1* and *pdi1*) and the ERAD pathway (*der1* and *hrd1*). As shown in Fig. 8A, the transcript level of *bip1* was slightly increased in the QGOD, a glucose oxidase-expression strain, than the parental strain QM53, while that of *pdi1* was higher in QGOD than QM53 after 72 h induction, suggesting the UPR triggered in QGOD. In addition, the *der1* expression was similar between the two strains QGOD and QM53, while the *hrd1* gene was expressed at a lower level in QGOD than that in the QM53, indicating the ERAD was also reduced in QGOD (Fig. 8B). Similarly, expression of heterologous laccase A by replacing the CBH1 via HR could decrease the level of the ERAD in comparison with that by random insertion into the genome in previous study [41]. In our study, overexpression of *gox* at the *cbh1* locus (that is, deletion of CBH1) induced the slight ERAD (data not shown). It was also found that the transcript level of *gox* in QGOD was even higher than those of *cbh1* and *cbh2* in the parental stain QM53 (Fig. 8A, B). This observation was probably related to the absence of the endogenous CBH1 and CBH2 in QGOD, which might decrease the repression under secretion stress (RESS), a mechanism where an ER stress response was induced by down regulation of the transcript levels of the genes encoding major secreted proteins in filamentous fungi [44]. Thus, high-level expression of *gox* was presumed to be attributed to the remission of RESS after deletion of *cbh1* and *cbh2*.

Subsequently, the culture supernatants of QGOD were used to detect the production of glucose oxidase. SDS-PAGE analysis showed that a band of about 69 KDa was produced in QGOD while not observed in the parental strain QM53 (Fig. 8C). The band size of glucose oxidase was also reported by Belyad et al. [45]. Furthermore, the protein band was identified by mass spectrometry and confirmed to be glucose oxidase (data not shown). Meanwhile, the bands of CBH1 (about 66 kDa) and CBH2 (58 kDa) were shown in QM53, while disappeared in QGOD, indicating that CBH1 and CBH2 were removed in QGOD. Then, the production level of glucose oxidase by QGOD was determined using o-dianisidine as substrate. It was found that the glucose oxidase activity of QGOD reached a maximum of 43.77 U/mL in day 9 (Fig. 8D). These results suggested that glucose oxidase was successfully secreted into the fermentation broth in QGOD. It was reported that the *A. niger* glucose oxidase was expressed by construction of the AnGOx-expressing cassette randomly integrated into the genome of *T. reesei* and its production level was 0.6 U/mL, although the genes (*snc1*, *bip1* and *hac1*) in the secretion pathway was simultaneously stimulated [46]. More recently, a CRISPR/Cas9-mediated genome editing system was used to express the *A. niger* glucose oxidase in *T. reesei*, where one sgRNA was liberated for targeted integration of the AnGOx-expressing cassette into the *cel3c* (encodes a β-glucosidase) locus and the production level of glucose oxidase reached 309 U/mL [47]. However, a large number of endogenous cellulases were also present in the fermentation broth, which may need complicate process to purify the target glucose oxidase. In this study, a novel tRNA−gRNA array-based CRISPR−Cas9 system released four sgRNAs to facilitate deleting *cbh1/cbh2* and overexpressing the heterologous glucose oxidase, and the production level of glucose oxidase reached 43.77 U/mL with a relatively clear background in *T. reesei*.

## Conclusions

In summary, this study established a multi-sgRNA processing platform to improve the efficiency of the CRISPR−Cas9 system for homologous recombination and genetic manipulation in *T. reesei*. The system was successfully used to improve the production of cellulase and xylanase by overexpressing Xyr1-A824V at the *ace1* locus, as well as the production of the heterologous glucose oxidase by deleting the major cellualse genes *cbh1* and *cbh2*. This type of the tRNA−gRNA array-based CRISPR−Cas9 system is especially suitable for multiple gene manipulations. Taken together, the system is not only important for genome-wide metabolic engineering of *T. reesei* for the production of cellulase and recombinant protein, but also expands the CRISPR toolset for use in filamentous fungi.

## Material and methods

### Strains and culture conditions

*Escherichia coli* DH5α (Vazyme, China) was used as a routine cloning host and cultured in liquid Luria–Bertani broth containing ampicillin (100 μg/mL) at 37 ℃. *T. reesei* QM53, the *mus53*-deletion strain derived from QM9414 with improved integration frequency [48], was employed as the parental strain in this study. All *T. reesei* transformants were selected using the *ptrA* (pyrithiamine resistance gene) selection marker on Minimal Medium (MM) plates supplemented with pyrithiamine hydrobromide (0.5 μg/mL). In addition, the glucose oxidase-expressing transformants were transferred onto the plates where lactose served as the sole carbon source at 30℃ for 3 days, and then they were screened by dumping the liquid mixed with 1% agarose, 0.26 mM o-dianisidine dihydrochloride, 4% glucose and 4 U/mL horseradish peroxidase to test glucose oxidase. Approximately 0.5 cm^3^ of MM agar containing mycelia was inoculated on a solid plate containing dextrose agar (PDA), MM agar plate with the addition of 2‰ Triton-100X and replacement of 2.0% glucose 0.5% Avicel (cellulose) and 1.0% glucose, or MM agar plate with the addition of 0.5% Triton-100X and replacement of 2.0% glucose with 0.5% Avicel. *T. reesei* was routinely grown and maintained PDA plate for conidial germination at 30℃ for 7 days. The composition of the inducing fermentation medium was 20% carbon source (glucose, Avicel or lactose), 0.5% (NH_4_)_2_SO_4_, 2.0% corn steep liquor, 0.2% peptone, 1.5% KH_2_PO_4_, 0.06% MgSO_4_·7H_2_O, 0.06% CaCl_2_·2H_2_O, 10.0% trace element solution (0.005 mg/L FeSO_4_·7H_2_O, 0.0014 mg/L ZnSO_4_·2H_2_O, 0.002 mg/L CoCl_2_·6H_2_O, 0.0017 mg/L MnSO_4_·H_2_O). All *T. reesei* strains used are listed in Additional file 1: Table S1.

### Plasmid construction

#### Construction of the plasmid pFC33ptrA

For editing gene in this study, the *pyr*G marker was replaced by the *ptrA* marker in pFC330, an extra-chromosomal *cas9* expressing plasmid [26]. The 0.2 kb *tef1* terminator T*tef1* was amplified from the plasmid pFC330 using the primer pair Ttef1-210DF(Pml I)/Ttef1-489DR(ptrA). The *ptrA* expression cassette (2.1 kb) was obtained from the plasmid T-*ptrA* using the prime pair PtrA-F/PtrA-R(Nhe I). The above two fragments were fused through Double-joint PCR. Then the fused fragment was amplified by the primer pair Ttef1-210DF(Pml I)/PtrA-R(Nhe I) containing the *Pml* I and *Nhe* I restriction sites in 5 extremities, respectively. The fragment was digested with *Pml* I and *Nhe* I and inserted into the *Pml* I and *Nhe* I site of pFC330 [26], yielding the plasmid pFC33ptrA.

#### Construction of the plasmid pFC33ptrA-5StsgRNA

The sgRNA expression element 5StsgRNA was synthesized containing *T. reesei 5S rRNA* promoter [25], tRNA^(Gly)^ of *T. reesei*, the sgRNA scaffffolds and T_6_ terminator (Additional file 1: Table S4), which was inserted into the *Pml* I and *Nhe* I sites of plasmid pFC33ptrA, resulting in pFC33ptrA-5StsgRNA (Additional file 1: Fig. S1A).

#### Construction of the plasmid pFC33ptrA-5StsgRNA(cre1*2)

The plasmid pFC33ptrA-5StsgRNA(cre1*2) was achieved in two steps: at first, a sgRNA cassette 5StsgRNA(cre1*2) was assembled, including two sgRNAs (sgRNA(*cre1*U) and sgRNA(*cre1*D)) that both targeted *cre1*. This sgRNA cassette was assembled out of three different overlapping fragments: 5S rRNA*-*tRNA (597 bp), sgRNA(*cre1*U)-tRNA (187 bp), and sgRNA(*cre1*D)-tRNA-T_6_ (183 bp), which were amplified from p5StsgRNA using the primer pairs 5S rRNA-497-UF(Bgl II)/tRNA(cre1U)-R, cre1U-sgRNA-F/tRNA(cre1D)-R (187 bp) and cre1D-sgRNA-F/T6-R(Pac I), respectively. Then, 5StsgRNA(cre1*2) was sub-cloned into pFC33ptrA between the *Bgl* II and *Pac*I unique restriction sites. The resulting vector was named as pFC33ptrA-5StsgRNA(cre1*2).

#### Construction of the plasmid pFC33ptrA-5StsgRNA(ace1*2)

To construct the pFC33ptrA-5StsgRNA(ace1*2) plasmid, an sgRNA cassette 5StsgRNA(ace1*2) was first constructed, which carried two sgRNAs (sgRNA(*ace1*U) and sgRNA(*ace1*D)) both targeting *ace1*. This sgRNA cassette was assembled out of three different overlapping fragments: 5S rRNA−tRNA (597 bp), sgRNA(*ace1*U) −tRNA (187 bp), and sgRNA(*ace1*D)−tRNA-T_6_ (183 bp), which were amplified from p5StsgRNA using the primer pairs 5S rRNA-497-UF(Bgl II)/tRNA(ace1U)-R, ace1U-sgRNA-F/tRNA(ace1D)-R and ace1D-sgRNA-F/T6-R(Pac I), respectively. Then 5StsgRNA(ace1*2) was amplified by the primer pair 5S rRNA-497-UF(Bgl II)/T6-R(Pac I) and inserted into pFC33ptrA between the *Bgl* II and *Pac*I sites. The resulting vector was named as pFC33ptrA-5StsgRNA(ace1*2).

#### Construction of the plasmid pFC33ptrA-5StsgRNA(ace1)

To construct the pFC33ptrA-5StsgRNA(ace1) plasmid, an sgRNA cassette 5StsgRNA(ace1) was first constructed, which carried one sgRNA(sgRNA(*ace1*U) that targeted *ace1*. This sgRNA cassette was assembled out of two different overlapping fragments: 5S rRNA*-*tRNA (597 bp) and sgRNA(*ace1*U)-tRNA-T_6_ (183 bp), which were amplified from p5StsgRNA using the primer pairs 5S rRNA-497-UF(Bgl II)/tRNA(ace1U)-R, ace1U-sgRNA-F/T6-R(Pac I), respectively. Then 5StsgRNA(ace1) was amplified by the primer pair 5S rRNA-497-UF(Bgl II)/T6-R(Pac I) and inserted into pFC33ptrA between the *Bgl* II and *Pac*I sites. The resulting vectors was named pFC33ptrA-5StsgRNA(ace1).

#### Construction of the plasmid pFC33ptrA-5StsgRNA(cbh1*2-cbh2*2)

The 5StsgRNA(cbh1*2-cbh2*2) fragment contained four sgRNAs: two sgRNAs (sgRNA(cbh1U) and sgRNA(cbh1D)) targeting *cbh1* and two sgRNAs (sgRNA(cbh2U) and sgRNA(cbh2D)) targeting *cbh2.* They were first synthesized and inserted between the *Bgl* II and *Pac*I sites of the plasmid pFC33ptrA, yielding the new plasmid pFC33ptrA-5StsgRNA(cbh1*2-cbh2*2). The sequence information of the 5StsgRNA(cbh1*2-cbh2*2) fragment can be found in Supplementary Information (Additional file 1: Table S4). All the plasmids were confirmed by DNA sequencing. All the primers and plasmids used are listed in Additional file 1: Table S2 and Table S1.

### Design of sgRNAs

The online tool CRISPOR (http://crispor.tefor.net/crispor.py) was used to design the 20-bp protospacer for sgRNAs. All protospacer sequences used in this study are presented in Additional file 1: Table S3.

### Construction of donor DNA cassettes

All the expression cassettes were used as donor DNAs, which would be integrated at the target sites by homologous recombination that was mediated through the tRNA−gRNA array-based CRISPR−Cas9 editing system. To achieve overexpression of *xyr1-A824V* at the *ace1* locus, the 5’ upstream (U-*ace1*, 1.5 kb) and 3’ downstream (D-*ace1*, 1.5 kb) of the target gene *ace1* were amplified from the *T. reesei* genome with the primer pairs ace1-1591-UF/ace1-31UR and ace1-2372DF(TtrpC)/ace1-3912DR, respectively. The coding sequence of Xyr1-A824V (2.9 kb), a mutation of the main activator Xyr1 of glycoside hydrolases, was fused through Double-joint PCR by two DNA fragments, which were amplified from the *T. reesei* genome using the primer pairs xyr1-F/xyr1-A824V-UR (2.5 kb) and xyr1-A824V-DF/xyr1-R (0.4 kb), respectively. Then *xyr1-A824V* was further amplified using the prime pair xyr1-F/xyr1-R. The plasmid pAN7-1 was used as template to produce the *gpdA* promoter P*gpdA* and the *trpC* terminator T*trpC* using the primer pairs PgpdA-F(ace1U)/PgpdA-R(xyr1) (1.1 kb) and TtrpC-F(xyr1)/TtrpC-R pAB7-1 T*trpC* (0.7 kb), respectively [49]. All the above fragments were assembled as Fig. 3 and the resulting dDNA was named as dgXYR1-ace1. Then the *cdna1* promoter (1.1 kb) P*cdna1* was amplified from the *T. reesei* genome using the primer pair Pcdna1-F(ace1U)/Pcdna1-R(xyr1). And the dDNA dcXYR1-ace1 was further constructed by assembling the same fragments by replacing P*gpdA* with P*cdna1*.

To express the heterologous glucose oxidase via the CRISPR/Cas9 technology, the glucose oxidase expression cassette targeting the *cbh1* locus was constructed as donor DNA (dGOD-cbh1). The cassette consisted of three fragments. The first fragment containing the *cbh1* promoter P*cbh1* (1.4 kb) and the *cbh1* signal sequence (sp) was amplified from the *T. reesei* genome using the prime pair CBH1-1491UF/Pcbh1-R(Sp-cbh1). The second fragment was the codon-optimized *gox* gene of *A. niger* ATCC 9029 (GeneBank: EU532181.1, 1.8 kb) with a 6 × His tag, which was obtained with the primer pair Sp-cbh1 (gox)/GOD-R(6 × His) using the synthesized plasmid T-*gox* as template. And the third fragment is the *cbh1* terminator T*cbh1* (1.6 kb), which was amplified from the *T. reesei* genome using the prime pair Tcbh1-1641F (6 × His)/CBH1-1690DR. Meanwhile, the P*cbh1* and T*cbh1* sequences were also used as the 5’ and 3’ flanking fragments of *cbh1* for HR*.* All the above fragments were assembled as Fig. 7. All the primers used are listed in Additional file 1: Table S2.

### Transformation and strain construction

The CaCl_2_-mediated transformation of *T. reesei* protoplasts was carried out as described previously [50]. The dDNA dgXYR1-ace1 and dcXYR1-ace1 were co-transformed with pFC33ptrA-5StsgRNA (ace1*2) into *T. reesei* QM53 to generate the strains named as QA1Xg and QA1Xc, respectively. The pFC33ptrA-5StsgRNA (cbh1*2-cbh2*2) was co-transformed with dGOD-cbh1 into *T. reesei* QM53 and the resulting strain named as QGOD. These transformants were identified using PCR. And the PCR primers were designed to bind upstream or downstream of the genomic integration sites, as well as to amplify the sequences within the integrated locus.

### RT-qPCR analysis

Mycelia were collected for extraction of total RNA using the TRIzol™ Reagen (Invitrogen, USA). Subsequently, RNA was reversely transcribed into the complementary DNA (cDNA) using HiScript ® III RT SuperMix for qPCR (+ gDNA wiper) (Vazyme, China). Quantitative PCR was performed on LightCycler 480 System (Roche Diagnostics, Germany) and carried out with ChamQ Universal SYBR ®qPCR Master Mix (Vazyme, China). The transcript levels of all genes were quantified using the *actin* gene as the reference. The primers used in this study are listed in Additional file 1: Table S2.

### SDS-PAGE

Fermentation supernatants were mixed with loading buffer boiled at 100 ℃ for 5 min. Subsequently, the mixture and PageRuler Prestained Protein Ladder (Thermo Scientific, USA) were loaded onto SDS-PAGE gels prepared by 12.5% Non-Closure SDS-PAGE Color Preparation kit (Sangon Biotech, China).

### Enzyme assays

Filter paper activity (FPA), endoglucanase activity (EG) and xylanase activity were determined using the 3, 5-dinitrosalicylic acid (DNS) method to measure the amount of reducing sugar. FPA was measured using Whatman No. 1 paper as the substrate as described by Ghose (1987) [51]. EG and xylanase activity were measured using 1% carboxymethylcellulose (CMC-Na) and beech wood xylan (Yuanye, China) as substrate, respectively. Both reactions were mixed 2 mL of substrate and 0.5 mL of the sample in citrate buffer (pH 4.8) at 50 ℃ for 30 min, stopped by DNS and heated at 95 for 10 min. For determination of cellobiohydrolase activity (CBH), β-glucosidase activity (BGL) and β-xylosidase activity, 50 μL of 1 mg/mL p-nitrophenyl-D-cellobioside (pNPC, Sigma-Aldrich, China) and 10 mM p-nitrophenyl-β-D-glucopyranoside (pNPG, Sigma-Aldrich, China) were used as substrate and mixed with 100 μL diluted enzyme in sodium citrate buffer with pH of 4.8 and incubated at 50 ℃ for 30 min. Then the reaction was stopped by the addition of 150 μL of 10% Na_2_CO_3_ before measuring absorbance at 420 nm. One unit of enzyme activity is defined as the amount of enzyme that releases 1 μmol p-nitrophenol per minute. The protein concentration was determined by the Modified Bradford Protein Assay Kit (Sanggon Biotech, China) as indicated by manual instruction.

Glucose oxidase activity was measured based on the method in previous research [47]. In brief, the reaction system including 2.5 mL of 0.26 mM o-Dianisidine dihydrochloride (dissolved in sodium citrate buffer with pH of 6.0), 0.3 mL of 18% glucose and 0.1 mL of 100 U/mL horseradish peroxidase solution was mixed in the tube at 35℃ for 2 min. After adding 0.1 mL of diluted sample for 3 min, the reaction was terminated by 2 mL of 2 M sulfuric acid solution before measuring absorbance at 500 nm. One unit of glucose oxidase is defined as the amount of enzyme to oxidize 1.0 μmole of β-D-glucose to D-gluconolactone and H_2_O_2_ per minute in the assay conditions.

### Supplementary Information

**Additional file 1: Fig. S1.** Schematic structures of the plasmid pFC33ptrA-5StsgRNA and the tRNA^Gly^ precursor; **Fig. S2.** RT-qPCR analysis of the xylanolytic genes in the *T. reesei* strains QA1Xg, QA1Xc and the parental strain QM53; **Fig. S3.** PCR analysis of the gene deletion events of the related strains constructed by the CRISPR−Cas9 system; **Fig. S4.** PCR analysis of the loss of the pFC33ptrA-5StsgRNA (ace1*2) plasmid in *T. reesei* QA1Xg. **Table S1.** The* T. reesei* strains and related plasmids used in this study; **Table S2.** All primers used in this study; **Table S3.** Protospacers and protospacer adjacent motifs (PAMs) of the target genes used in this study; **Table S4.** The sequences of synthetic genes used in this study; **Table S5.** The efficiency of genome editing achieved in this study.

## Data Availability

All data generated or analyzed during this study are included in this published article and its Additional files.

## Abbreviations

CRISPR: Clustered regularly interspaced short palindromic repeats
Cas9: Protein 9 nuclease from *Streptococcus pyogenes*
sgRNA: Single-guide RNA
RNPs: Ribonucleoproteins
dDNA: Donor DNA
HR: Homologous recombination
NHEJ: Non-homologous end-joining
DSB: Double-strand break
PAM: Protospacer adjacent motif
FPA: Filter paper activity
CCR: Carbon catabolite repression
