# Supplementary material for: An efficient CRISPR/Cas9 genome editing system based on a multiple sgRNA processing platform in Trichoderma reesei for strain improvement and enzyme production
Source: Biotechnol Biofuels Bioprod. 2024 Feb 11;17:22. doi: 10.1186/s13068-024-02468-7 (PMC10859021; doi:10.1186/s13068-024-02468-7)
Supplement: Supplementary file 1 — Additional file 1: Fig. S1. Schematic structures of the plasmid pFC33ptrA-5StsgRNA and the tRNAGly precursor; Fig. S2. RT-qPCR analysis of the xylanolytic genes in the T. reesei strains QA1Xg, QA1Xc and the parental strain QM53; Fig. S3. PCR analysis of the gene deletion events of the related strains constructed by the CRISPR−Cas9 system; Fig. S4. PCR analysis of the loss of the pFC33ptrA-5StsgRNA (ace1*2) plasmid in T. reesei QA1Xg. Table S1. The T. reesei strains and related plasmids used in this study; Table S2. All primers used in this study; Table S3. Protospacers and protospacer adjacent motifs (PAMs) of the target genes used in this study; Table S4. The sequences of synthetic genes used in this study; Table S5. The efficiency of genome editing achieved in this study. [file 13068_2024_2468_MOESM1_ESM.doc]

### Supplementary Information

#
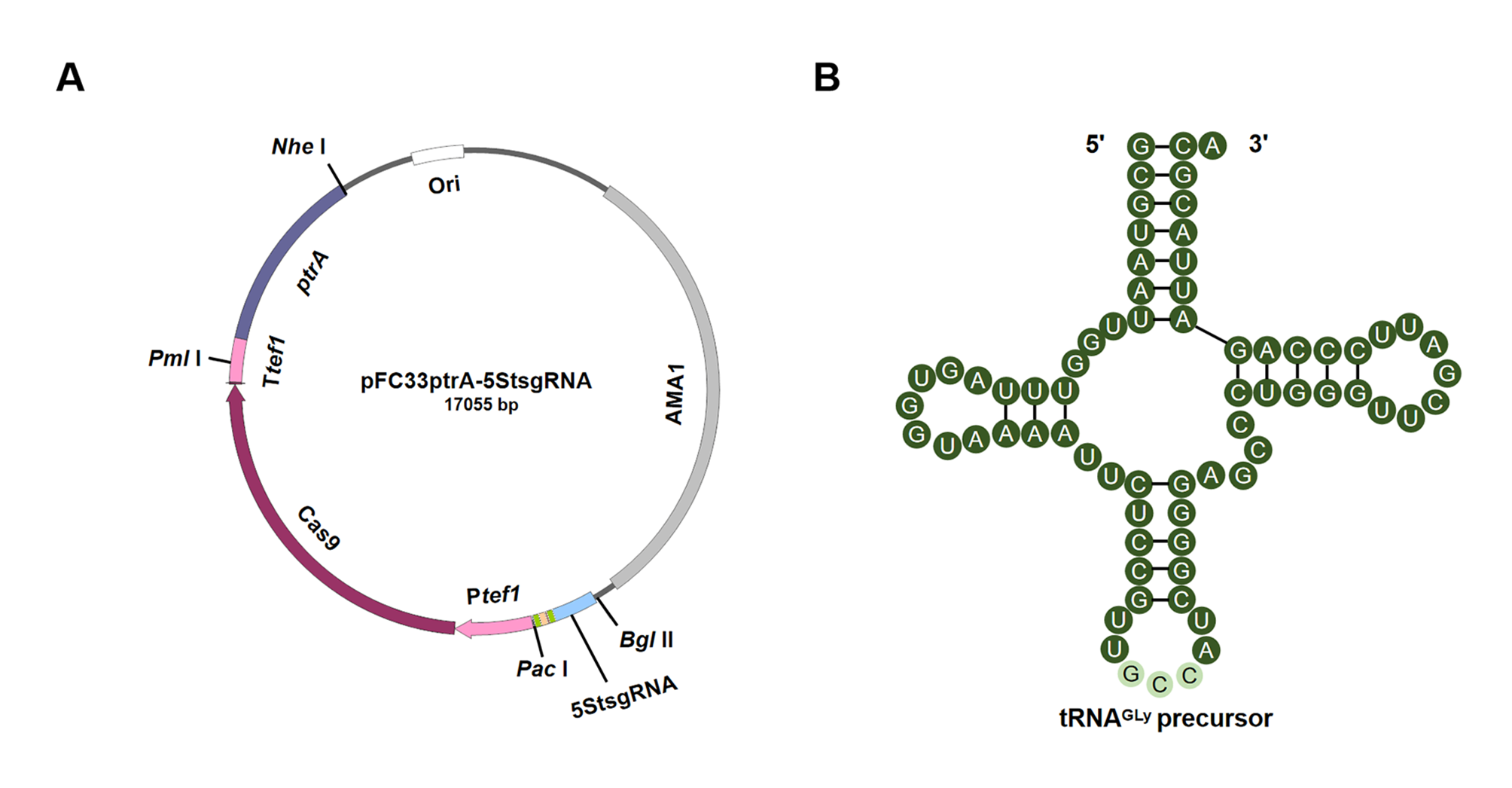
Fig. S1 Schematic structures of the plasmid pFC33ptrA-5StsgRNA and the tRNA^Gly^ precursor. (A) The plasmid map of the initiate sgRNA vector named pFC33ptrA-5StsgRNA. (B) The schematic diagrams of the secondary structure of the tRNA^Gly^ precursor.

**
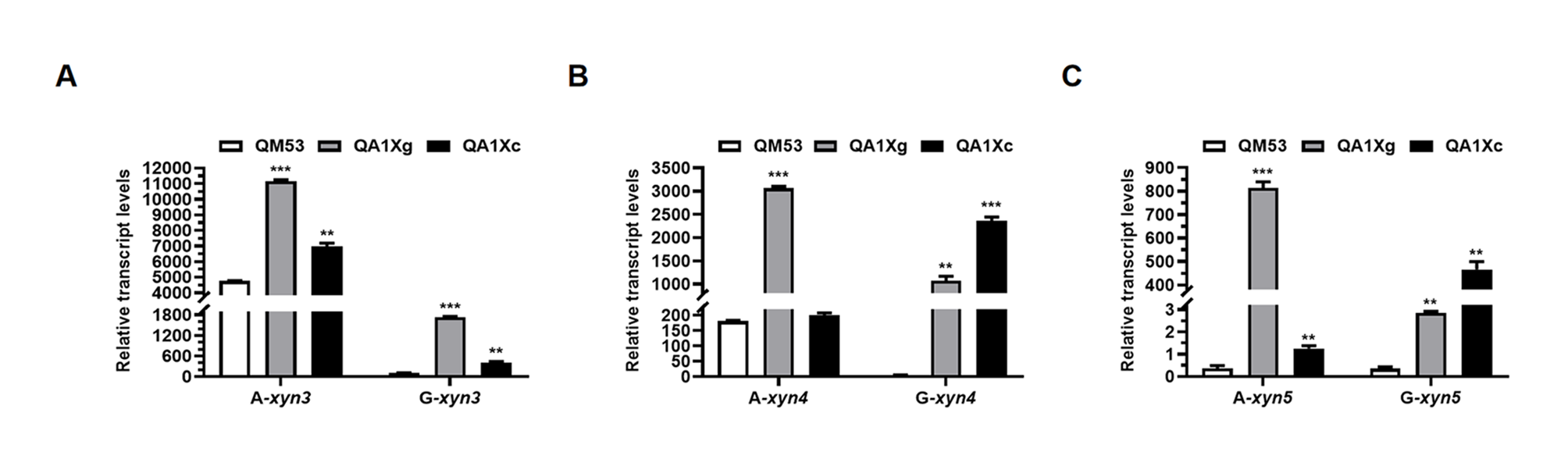
Fig. S2** RT-qPCR analysis of the xylanolytic genes in the *T. reesei* strains QA1Xg, QA1Xc and the parental strain QM53. Transcript levels of *xyn3* (A), *xyn4* (B) and *xyn5* (C). The alphabet A indicates the Avicel culture condition while G indicates the glucose culture conditions. Data are the Mean ± SD of the results from three independent experiments. All values were normalized to the *actin* expression under the same condition. Significant differences were analyzed using *t*-test. (*p < 0.05, **p < 0.01, ***p < 0.001.)

**
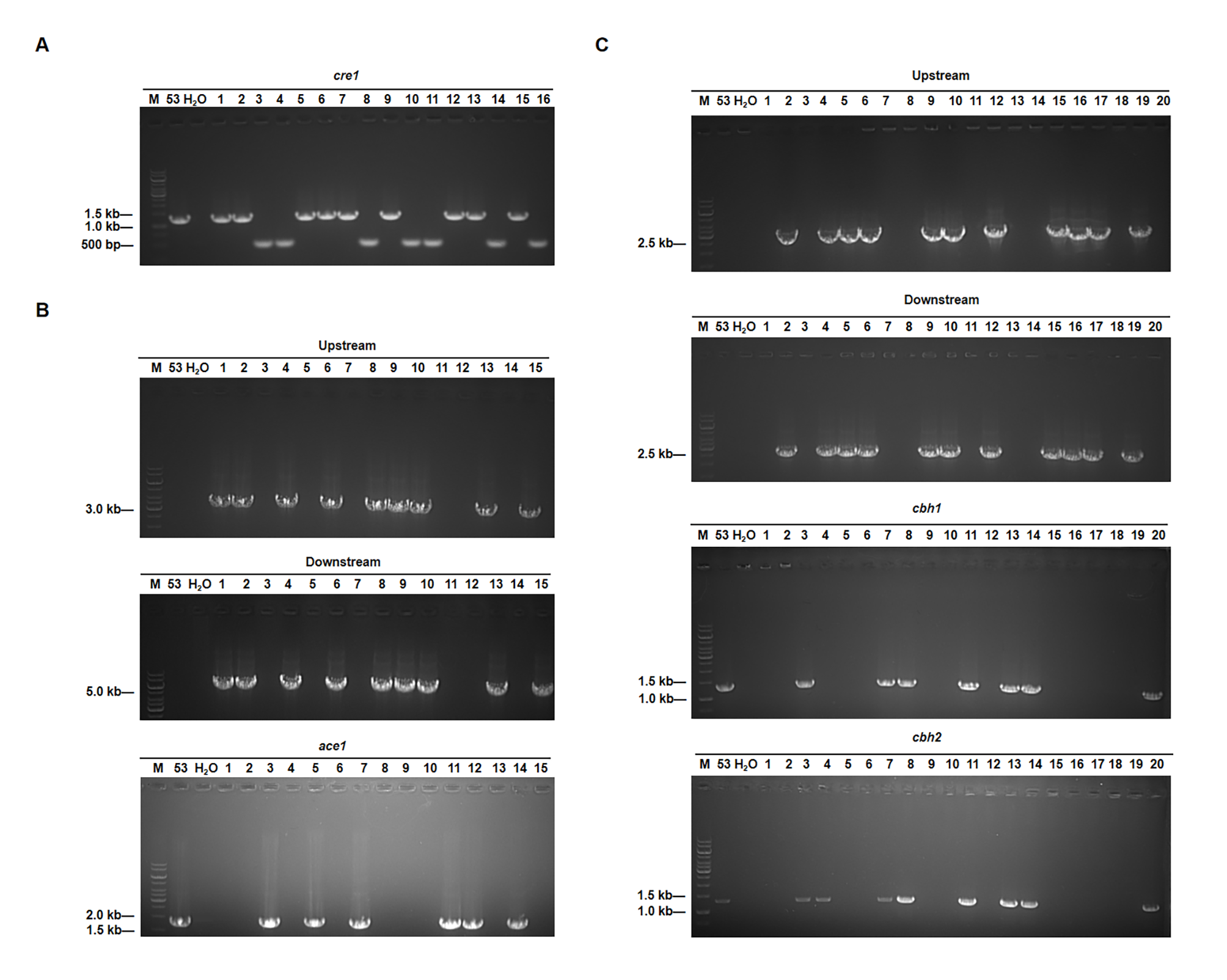
Fig. S3** PCR analysis of the gene deletion events of the related strains constructed by the CRISPR-Cas9 system. (A) Screening of the *cre1* deletion strains with the primer pair Y-cre1-270UF/Y-cre1-1067R binding externally to the two sgRNAs target loci. The PCR band with the size of 0.52 kb was expected in the Δ*cre1* strain QMC1, while that of 1.33 kb product in the parental strain QM53. M: DNA molecular mass marker; 53: the QM53 strain; H_2_O: the negative control; lanes 1-16: the randomly selected transformants and 7 out of 16 (43.8%) transformants were the targeted Δ*cre1* strains. (B) Screening of the strains containing the *xyr1-A824V* expression cassette replacement at the *ace1* locus. Upstream indicated that the 3.5-kb upstream fragment of the *xyr1-A824V* expression cassette was amplified using the primer pair Y-ace1-1768UF/Y-xyr1-593R from 9 out of 15 transformants (60.0%). Downstream indicated that the 5.2-kb downstream fragment of the *xyr1-A824V* expression cassette was amplified using the primer pair Y-ace1-4078DR/Y-xyr1-213F from 9 out of 15 (60.0%) transformants . The *ace1* result indicated that the 1.8-kb internal fragment of the *ace1* gene was amplified using primer pairs Y-ace1-411F/Y-ace1-2287R from 9 out of 15 transformants (60.0%). M: DNA molecular mass marker; 53: the QM53 strain; H_2_O: the negative control; lanes 1-15: the randomly selected transformants. (C) Screening of the strains containing the dGOD-cbh1 cassette replacement at the *cbh1* locus and the simultaneous deletion of *cbh2*. Upstream indicated that the 2.6-kb upstream fragment of the *gox* expression cassette was amplified using the primer pair Y-CBH1-1699UF/Y-GOD-972R from 11 out of 20 (55.0%) transformants. Downstream indicated that the 2.6-kb upstream fragment of the *gox* expression cassette was amplified using the primer pair Y-GOD-991F/Y-CBH1-3465DR from 11 out of 20 (55.0%) transformants. The *cbh1* result indicated that the 1.3-kb internal fragment of the *cbh1* gene was not amplified using the primer pair Y-cbh1-95F/Y-cbh1-1460R from 13 out of 20 (65.0%) transformants. The *cbh2* result indicated that the 1.3-kb internal fragment of the *cbh2* gene was not amplified using the primer pair Y-cbh2-206F/Y-cbh2-1511R from 12 out of 20 (60.0%) transformants. M: DNA molecular mass marker; 53: the QM53 strain; H_2_O: the negative control; lanes 1-20: the randomly selected transformants. Note: the strains containing the simultaneous deletion of *cbh1* and *cbh2* were 12 out of 20 (60.0%) transformants.

**
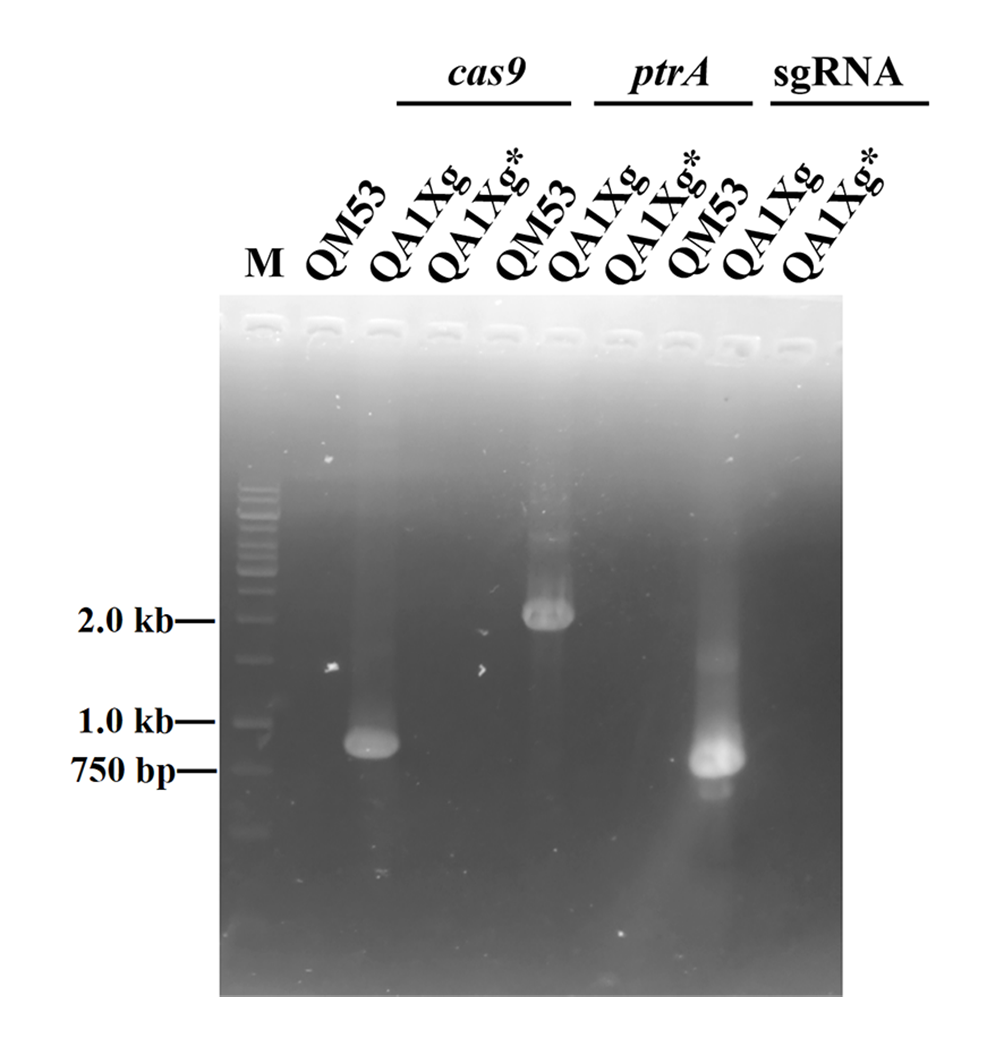
**

**Fig. S4** PCR analysis of the loss of the pFC33ptrA-5StsgRNA(ace1*2) plasmid in *T. reesei* QA1Xg. *Cas9*, *ptrA* and sgRNA indicate the amplified products using the prime pairs Y-330Cas9-3186F/Y-330Cas9-4080R, PtrA-F/PtrA-R(Nhe Ⅰ) and Y-sg-F/Y-sg-F, respectively. QA1Xg* indicates the colony of QA1Xg obtained after two generations of cultivation on the nonselective medium (the MM plate without pyrithiamine hydrobromide). QM53 is the parental strain.

**Table S1 The *T. reesei* strains and related plasmids used in this study**

| **Strain/Plasmid** | **Description** | **Source** |
| --- | --- | --- |
| **Strain** |  |  |
| QM53 | mus53-deletion strain derived from *T. reesei* QM9414 | Stored in our lab |
| QMC1 | Deletion *cre1* in QM53 | This study |
| QA1Xg | *ace1* replaced by *xyr1-A824V* under the control of *gpdA* promoter in QM53 | This study |
| QA1Xc | *ace1* replaced by *xyr1-A824V* under the control of *cdna1* promoter in QM53 | This study |
| QGOD | *cbh1* replaced by *gox* and deletion *cbh2* in QM53 | This study |
| **Plasmid** |  |  |
| pAN7-1 | *gpdA* promoter and *trpC* terminator | Punt et al. 1992 |
| T-*ptrA* | *ptrA* expression cassette | Stored in our lab |
| T-*gox* | codon-optimized *gox* gene from *A. niger* ATCC 9029 (GeneBank: EU532181.1) | This study |
| pFC330 | Extra-chromosomal *cas9* expressing plasmid with *pyrG* as marker | Nødvig et al. 2015 |
| pFC33ptrA | *pyrG* marker replaced by *ptrA* marker in pFC330 | This study |
| p5StsgRNA | containing sgRNA expression elements (*T. reesei 5S rRNA* promoter, tRNA^(Gly)^ of *T. reesei*, the sgRNA scaffffolds and T_6_ terminator) | This study |
| pFC33ptrA-5StsgRNA(cre1*2) | Extra-chromosomal *cas9* expressing plasmid with *ptrA* as marker and two sgRNAs targeting *cre1* locus | This study |
| pFC33ptrA-5StsgRNA(ace1*2) | Extra-chromosomal *cas9* expressing plasmid with *ptrA* as marker and two sgRNAs targeting *ace1* locus | This study |
| pFC33ptrA-5StsgRNA(cbh1*2-cbh2*2) | Extra-chromosomal *cas9* expressing plasmid with four sgRNAs targeting *cbh1* and *cbh2* locus using *ptrA* as marker | This study |

**Table S2 All primers used in this study**

| **Name** | **Nucleotide sequence (5’ to 3’)** | **Purpose** |
| --- | --- | --- |
| Ttef1-210DF(Pml Ⅰ) | ACGCACGTGATATTACATGACCTGG | *tef1* terminator |
| Ttef1-489DR(ptrA) | ATAAAGTGTAAAGCCTGGGGGTATTGGGATGAATTTTGTATG | *tef1* terminator |
| PtrA-F | CCCCAGGCTTTACACTTTAT | *ptrA* expression cassette |
| PtrA-R(Nhe Ⅰ) | CTAGCTAGCCCGCTCTTGCATCTTTGTT | *ptrA* expression cassette |
| 5S rRNA-497-UF(Bgl Ⅱ) | GGAAGATCTcatggtcatagctgtttccgctgagggtGACGTTGATGAGC | 5StsgRNA(cre1*2) expression cassette |
| tRNA(cre1U)-R | TGGATTTTTCCAACCTCCTGTGCGTAATCTGGGAATCG | 5StsgRNA(cre1*2) expression cassette |
| cre1U-sgRNA-F | CAGGAGGTTGGAAAAATCCAGTTTTAGAGCTAGAAATAGCAAGTT | 5StsgRNA(cre1*2) expression cassette |
| tRNA(cre1D)-R | ACCCCGGATCACACTCCCATTGCGTAATCTGGGAATCG | 5StsgRNA(cre1*2) expression cassette |
| cre1D-sgRNA-F | ATGGGAGTGTGATCCGGGGTGTTTTAGAGCTAGAAATAGCAAGTT | 5StsgRNA(cre1*2) expression cassette |
| T6-R(Pac Ⅰ) | CCTTAATTAAAAAAAATGCGTAATCTG | 5StsgRNA(cre1*2) expression cassette |
| Y-sg-F | TGCCTCTGGTCAGTTGGTCT | sgRNA verification |
| Y-sg-R | ACTTGGGCGGTGATTCTG | sgRNA verification |
| Y-cre1-270UF | CCTCACATCGCATCTCG | *cre1* deletion analysis |
| Y-cre1-1067R | TCGGGGCGGCTAATGAT | *cre1* deletion analysis |
| 5S rRNA-497-UF(Bgl Ⅱ) | GGAAGATCTcatggtcatagctgtttccgctgagggtGACGTTGATGAGC | 5StsgRNA(ace1*2) expression cassette |
| tRNA(ace1U)-R | CCGCAGAAGGACACCGGTGATGCGTAATCTGGGAATCG | 5StsgRNA(ace1*2) expression cassette |
| ace1U-sgRNA-F | TCACCGGTGTCCTTCTGCGGGTTTTAGAGCTAGAAATAGCAAGTT | 5StsgRNA(ace1*2) expression cassette |
| tRNA(ace1D)-R | GTCATTGTTTACCGATATGCTGCGTAATCTGGGAATCG | 5StsgRNA(ace1*2) expression cassette |
| ace1D-sgRNA-F | GCATATCGGTAAACAATGACGTTTTAGAGCTAGAAATAGCAAGTT | 5StsgRNA(ace1*2) expression cassette |
| T6-R(Pac Ⅰ) | CCTTAATTAAAAAAAATGCGTAATCTG | 5StsgRNA(ace1*2) expression cassette |
| ace1-1591-UF | TAGTTGAGCGGCTTTGTT | *ace1* upstream homology arm |
| ace1-31UR | TGGGTGGGGAAGATGTATG | *ace1* upstream homology arm |
| Pcdna1-F(ace1) | CATACATCTTCCCCACCCAGAATTCGGTCTGAAGGACG | *cdna1* promoter |
| Pcdna1-R(xyr1) | ACGGAGAGGATTGGACAACATGTTGAGAGAAGTTGTTGGATTG | *cdna1* promoter |
| PgpdA-F(ace1) | CATACATCTTCCCCACCCAAGACCTAATACAGCCCCTACAA | *gpdA* promoter |
| PgpdA-R(xyr1) | ACGGAGAGGATTGGACAACATTGTCTGCTCAAGCGGGGTA | *gpdA* promoter |
| xyr1-F | ATGTTGTCCAATCCTCTCCGT | *xyr1-A824V* |
| xyr1-A824V-UR | GACAGCCGATACCGCGT | *xyr1-A824V* |
| xyr1-A824V-DF | GACGAGCCACGCGGTATCGGCTGTCGAAGCTATTAGCCAGATTCTCGA | *xyr1-A824V* |
| xyr1-R | TTAGAGGGCCAGACCGGTT | *xyr1-A824V* |
| TtrpC-F(xyr1) | TAACGGAACCGGTCTGGCCCTCTAAGGATCCACTTAACGTTACTGAA | *trpC* terminator |
| TtrpC-R | TTAGAGGGCCAGACCGGTT | *trpC* terminator |
| ace1-2372DF(TtrpC) | GCGCCCACTCCACATCTCCACTCGACAAACGCTTATCCACTCG | *ace1* downstream homology arm |
| ace1-3912DR | CCTTTTGAGCAGTGGACG | *ace1* downstream homology arm |
| Y-ace1-1768UF | GGGCATTTTTTTTGGGGAT | Verification of the *xyr1-A824V* overexpression strain |
| Y-xyr1-593R | GAAGATTCGCTTTGCTGGC | Verification of the *xyr1-A824V* overexpression strain |
| Y-xyr1-213F | CGCCAGGGCTCTCTTATTG | Verification of the *xyr1-A824V* overexpression strain |
| Y-ace1-4078DR | CTTCACGAAATCCTTACCGA | Verification of the *xyr1-A824V* overexpression strain |
| Y-ace1-411F | CGACGCACCAGAAACCA | *ace1* deletion analysis |
| Y-ace1-2287R | GGTTGAAGATGTCGGGC | *ace1* deletion analysis |
| CBH1-1491UF | TTGTGAAGTCGGTAATCCC | *cbh1* promoter (including the *cbh1* signal peptide) (upstream homology arm) |
| Pcbh1-R(Sp-cbh1) | AGATGACGGCCAACTTCCGATACATTGGGTTTCTGTGCCTCAA | *cbh1* promoter (including the *cbh1* signal peptide) (upstream homology arm) |
| Sp-cbh1(gox) | ATGTATCGGAAGTTGGCCGTCATCTCGGCCTTCTTGGCCACAGCTCGTGCTATGCAGACTCTCCTTGTGAGCT | *gox* (including His-tag) |
| GOD-R(6xHis) | CTAATGATGATGATGATGATGCTGCATGGAAGCATAATCTTCC | *gox* (including His-tag) |
| Tcbh1-1641F(6XHis) | CATCATCATCATCATCATTAGAGGTCCTGAACCCTTACTAC | *cbh1* terminator (downstream homology arm) |
| CBH1-1690DR | ATTCCCCATTCAAGTCAG | *cbh1* terminator (downstream homology arm) |
| Y-CBH1-1699UF | GGGTTTGGAGCAATGTGG | Verification of the *gox* expression strain |
| Y-GOD-972R | GGATGGACTTCATTCCGATA | Verification of the *gox* expression strain |
| Y-GOD-991F | CTGGAGCCCCTTGGTAT | Verification of the *gox* expression strain |
| Y-CBH1-3465DR | TCTCCTATGTCTGCTCGG | Verification of the *gox* expression strain |
| Y-cbh1-95F | ATGGCAGAAATGCTCGTC | *cbh1* deletion analysis |
| Y-cbh1-1460R | AATGGGTCCGAACTTGAT | *cbh1* deletion analysis |
| Y-cbh2-206F | ACTCCAACGACTATTACTC | *cbh2* deletion analysis |
| Y-cbh2-1511R | CAGTGGGAGTCAAATCGT | *cbh2* deletion analysis |
| Y-330Cas9-3186F | ATTGAGACCAACGGCGAGAC | *cas9* verification |
| Y-330Cas9-4080R | GATACGGGTTTCATAGAGCC | *cas9* verification |
| Actin-qF | CCCAAGTCCAACCGTGAGA | RT-qPCR for *actin* |
| Actin-qR | CAATGGCGTGAGGAAGAGC | RT-qPCR for *actin* |
| Xyr1-qF | TCTTCTACGGCGTCTATCTCC | RT-qPCR for *xyr1* |
| Xyr1-qR | GTGTGCCCTAACAATGGTCTC | RT-qPCR for *xyr1* |
| Cbh1-qF | GCGGCATGGTTCTGGTCA | RT-qPCR for *cbh1* |
| Cbh1-qR | TCGTTTGTCGGGTAGGTGGA | RT-qPCR for *cbh1* |
| Cbh2-qF | CTGGTCCAACGCCTTCTTCA | RT-qPCR for *cbh2* |
| Cbh2-qR | GACCCAGACAAACGAATCCAG | RT-qPCR for *cbh2* |
| Egl1-qF | CGGCTACAAAAGCTACTACG | RT-qPCR for *egl1* |
| Egl1-qR | CTGGTACTTGCGGGTGAT | RT-qPCR for *egl1* |
| Egl2-qF | ACGAGCCTTTGGTCGCAGTT | RT-qPCR for *egl2* |
| Egl2-qR | GGCAGCCCAGGTGTTGATGT | RT-qPCR for *egl2* |
| Bgl1-qF | AGTGACAGCTTCAGCGAG | RT-qPCR for *bgl1* |
| Bgl1-qR | GGAGAGGCGTGAGTAGTTG | RT-qPCR for *bgl1* |
| Xyn1-QF | CAGCTATTCGCCTTCCAACAC | RT-qPCR for *xyn1* |
| Xyn1-Qr | CAAAGTTGATGGGAGCAGAAG | RT-qPCR for *xyn1* |
| Xyn2-QF | GGTCCAACTCGGGCAACTTT | RT-qPCR for *xyn2* |
| Xyn2-Qr | CCGAGAAGTTGATGACCTTGTTC | RT-qPCR for *xyn2* |
| XYN3-QF | CACCGACGACGGATTACA | RT-qPCR for *xyn3* |
| XYN3-QR | GTAAGATGCCAACAATGC | RT-qPCR for *xyn3* |
| XYN4-QF | TTGCTATTCCCGTCATCA | RT-qPCR for *xyn4* |
| XYN4-QR | CCAAAAAGTCTTCATCGC | RT-qPCR for *xyn4* |
| XYN5-QF | CTACGGCTGGAGCACTAACC | RT-qPCR for *xyn5* |
| XYN5-QR | CTGGTGACGCTGCCCTTC | RT-qPCR for *xyn5* |
| GOX-QF | CCGCAACATCTCCAACTC | RT-qPCR for *gox* |
| GOX-QR | ACCATCAATGACACGCAG | RT-qPCR for *gox* |
| PDI1-QF | GTTGTCGTTGCCCACTCTTAC | RT-qPCR for *pdi1* |
| PDI1-QR | AGTCGCTCTTGGCATACAGG | RT-qPCR for *pdi1* |
| BIP1-QF | GATGCCAACGGTATCCTCA | RT-qPCR for *bip1* |
| BIP1-QR | TGCGGTCAATCTCCTCCT | RT-qPCR for *bip1* |
| HRD1-QF | CCCAATGATGCCAAACTG | RT-qPCR for *hrd1* |
| HRD1-QR | TGCTGCTTTCAGGTGGAG | RT-qPCR for *hrd1* |
| DER1-QF | CCTCGTTTACATTTGGTCTCG | RT-qPCR for *der1* |
| DER1-QR | GCCCATGATCTCATCCCTC | RT-qPCR for *der1* |

**Table S3 Protospacers and protospacer adjacent motifs (PAMs) of the target genes used in this study**

| **Gene** | **Protospacer (5’ to 3’)** | **PAM** | **sgRNA** |
| --- | --- | --- | --- |
| *cre1* | CAGGAGGTTGGAAAAATCCA | CGG | sgRNA(cre1U) |
| *cre1* | ATGGGAGTGTGATCCGGGGT | GGG | sgRNA(cre1D) |
| *ace1* | TCACCGGTGTCCTTCTGCGG | GGG | sgRNA(ace1U) |
| *ace1* | GCATATCGGTAAACAATGAC | TGG | sgRNA(ace1D) |
| *cbh1* | GGCCACAGCTCGTGCTCAGT | CGG | sgRNA(cbh1U) |
| *cbh1* | AGCTTCCAGTGGTAGTGGCT | GGG | sgRNA(cbh1U) |
| *cbh2* | ACACTGGGAGTAATAGTCGT | TGG | sgRNA(cbh1D) |
| *cbh2* | CGTTGCTGGATTCGTTTGTC | TGG | sgRNA(cbh1D) |

**Table S4 The sequences of synthetic genes used in this study**

| **Name** | **DNA Sequence (5’ to 3’)** |
| --- | --- |
| 5StsgRNA | GACGTTGATGAGCTCAAGAGCCGTGAGAAGGAAATCGTTGGGGGCCCTCCTGGAACCGGCGAGCTGTGGCTCAGCTTCTCGGGATTCCCGCGATACATTGGGGGCAACCTTCTGCCTGGTGCGTAAGAAGCTGGTATGACCGAGCTTAAGCCAGGGATATCTATTGATGAACAGGACGCTCGACTATGGGACTTTACCAAAGAAAAGATGCCGGTCTCCAGTAGCGCGTGGGCGAGAGGAGCAGAGACACTCTAGATAGCACTACAATGAACATTCATCTCTCAACCCAACTTGTCAACTCTACCAACCAACGAATCAACCAACCTTTTTTTCTCCTCAGATCCTCCCATTCATGATCGTAGACAAGACACAGCCCATCACATACGACCACAGGGTGTGGAAAACAGGGCTTCCCGTCCGCTCAGCCGTACTTAAGCCACACGCCGGGAGGTTAGTAGTTGGGTGGGTGACCACCAGCGAATCCCTTCTGTTGTATGGCGTAATTGGTTTAGTGGTAAAATTCTCCGTTGCCATCGGGGAGCCCTGGGTTCGATTCCCAGATTACGCAGTTTTAGAGCTAGAAATAGCAAGTTAAAATAAGGCTAGTCCGTTATCAACTTGAAAAAGTGGCACCGAGTCGGTGCGCGTAATTGGTTTAGTGGTAAAATTCTCCGTTGCCATCGGGGAGCCCTGGGTTCGATTCCCAGATTACGCATTTTTT |
| 5StsgRNA(cbh1*2-cbh2*2) | GACGTTGATGAGCTCAAGAGCCGTGAGAAGGAAATCGTTGGGGGCCCTCCTGGAACCGGCGAGCTGTGGCTCAGCTTCTCGGGATTCCCGCGATACATTGGGGGCAACCTTCTGCCTGGTGCGTAAGAAGCTGGTATGACCGAGCTTAAGCCAGGGATATCTATTGATGAACAGGACGCTCGACTATGGGACTTTACCAAAGAAAAGATGCCGGTCTCCAGTAGCGCGTGGGCGAGAGGAGCAGAGACACTCTAGATAGCACTACAATGAACATTCATCTCTCAACCCAACTTGTCAACTCTACCAACCAACGAATCAACCAACCTTTTTTTCTCCTCAGATCCTCCCATTCATGATCGTAGACAAGACACAGCCCATCACATACGACCACAGGGTGTGGAAAACAGGGCTTCCCGTCCGCTCAGCCGTACTTAAGCCACACGCCGGGAGGTTAGTAGTTGGGTGGGTGACCACCAGCGAATCCCTTCTGTTGTATGGCGTAATTGGTTTAGTGGTAAAATTCTCCGTTGCCATCGGGGAGCCCTGGGTTCGATTCCCAGATTACGCAGGCCACAGCTCGTGCTCAGTGTTTTAGAGCTAGAAATAGCAAGTTAAAATAAGGCTAGTCCGTTATCAACTTGAAAAAGTGGCACCGAGTCGGTGCGCGTAATTGGTTTAGTGGTAAAATTCTCCGTTGCCATCGGGGAGCCCTGGGTTCGATTCCCAGATTACGCAAGCTTCCAGTGGTAGTGGCTGTTTTAGAGCTAGAAATAGCAAGTTAAAATAAGGCTAGTCCGTTATCAACTTGAAAAAGTGGCACCGAGTCGGTGCGCGTAATTGGTTTAGTGGTAAAATTCTCCGTTGCCATCGGGGAGCCCTGGGTTCGATTCCCAGATTACGCAACACTGGGAGTAATAGTCGTGTTTTAGAGCTAGAAATAGCAAGTTAAAATAAGGCTAGTCCGTTATCAACTTGAAAAAGTGGCACCGAGTCGGTGCGCGTAATTGGTTTAGTGGTAAAATTCTCCGTTGCCATCGGGGAGCCCTGGGTTCGATTCCCAGATTACGCACGTTGCTGGATTCGTTTGTCGTTTTAGAGCTAGAAATAGCAAGTTAAAATAAGGCTAGTCCGTTATCAACTTGAAAAAGTGGCACCGAGTCGGTGCGCGTAATTGGTTTAGTGGTAAAATTCTCCGTTGCCATCGGGGAGCCCTGGGTTCGATTCCCAGATTACGCATTTTTT |

Note: Black letters indicate the *5S rRNA* promoter from *T. reesei* for sgRNA expression. Purple letters indicate the protospacers. Yellow letters indicate the T_6_ terminator. Red letters indicate sgRNA scaffold natural sequence from *Streptococcus pyogene*. Green letters indicate the tRNA sequence that coding glycine from *T. reesei*.

**Table S5 The efficiency of genome editing achieved in this study**

| gene | sgRNA | dDNA | editing size | editing efficiency |
| --- | --- | --- | --- | --- |
| *cre1* | sgRNA(cre1U); sgRNA(cre1D) |  | 818 bp | 43.75% (7/16) |
| *ace1* | sgRNA(ace1U); sgRNA(ace1D) | dgXYR1-ace1/  dcXYR1-ace1 | 2403 bp | 60.00% (9/15) |
| *cbh1* and *cbh2* | sgRNA(cbh1U); sgRNA(cbh1D);  sgRNA(cbh2U); sgRNA(cbh2D) | dGOD-cbh1 | *cbh1*: 1590bp  *cbh2*: 1225bp | 60.00% (12/20) |

### Supporting references

Nødvig CS, Nielsen JB, Kogle ME, Mortensen UH. A CRISPR-Cas9 System for genetic engineering of filamentous fungi. PLoS One. 2015;10 (7): e0133085.

Punt PJ, Kramer C, Kuyvenhoven A, Pouwels PH, van den Hondel CA. An upstream activating sequence from the *Aspergillus nidulans* *gpdA* gene. Gene. 1992;120 (1): 67-73.
